# Human Milk-Based or Bovine Milk-Based Fortifiers Differentially Impact the Development of the Gut Microbiota of Preterm Infants

**DOI:** 10.3389/fped.2021.719096

**Published:** 2021-11-30

**Authors:** Miriam Aguilar-Lopez, Christine Wetzel, Alissa MacDonald, Thao T. B. Ho, Sharon M. Donovan

**Affiliations:** ^1^Division of Nutritional Sciences, University of Illinois Urbana-Champaign, Urbana, IL, United States; ^2^Carle Hospital, Urbana, IL, United States; ^3^Department of Pediatrics, Morsani College of Medicine, University of South Florida, Tampa, FL, United States

**Keywords:** gut microbiota, milk fortifiers, preterm infant, growth, enterotype

## Abstract

**Background:** Preterm infants are exposed to different dietary inputs during their hospitalization in the neonatal intensive care unit (NICU). These include human milk (HM), with a human milk-based (HMF) or a bovine milk-based (BMF) fortifier, or formula. Milk consumption and the type of fortification will cause changes in the gut microbiota structure of preterm infants. This study aimed to characterize the gut microbiota of PT infant according to the type of feeding and the type of HM fortification and its possible association with infant's growth.

**Methods:** Ninety-seven infants born ≤33 wks of gestation or <1,500 g were followed during the hospitalization period in the NICU after birth until discharge. Clinical and dietary information was collected, including mode of delivery, pregnancy complications, mechanical ventilation, use of antibiotics, weight, and type and amount of milk consumed. To characterize the gut microbiota composition, weekly stool samples were collected from study participants. The V3–V4 region of the 16S rRNA bacterial gene was Sequenced using Illumina MiSeq technology.

**Results:** After birth, black maternal race, corrected gestational age (GA) and exposure to pregnancy complications, had a significant effect on gut microbial diversity and the abundance of *Enterococcus, Veillonella, Bifidobacterium, Enterobacter*, and *Bacteroides*. Over the course of hospitalization, corrected GA and exposure to chorioamnionitis remained to have an effect on gut microbial composition. Two different enterotypes were found in the gut microbiota of preterm infants. One enriched in *Escherichia-Shigella*, and another enriched in uncharacterized *Enterobacteriaceae, Klebsiella* and *Clostridium sensu stricto 1*. Overall, HM and fortification with HMF were the most common feeding strategies. When consuming BMF, PT infants had higher growth rates than those consuming HMF. Milk and type of fortification were significantly associated with the abundance of *Clostridium sensu stricto 1, Bifidobacterium* and *Lactobacillus*.

**Conclusions:** This observational study shows the significant association between milk consumption and the exposure to HMF or BMF fortification in the fecal microbiota composition of preterm infants. Additionally, these results show the effect of other perinatal factors in the establishment and development of PT infant's gut microbiota.

## Introduction

The development of the gut microbiota during the neonatal period plays a key role in the characteristics of this ecosystem later in life and affects growth, immune and cognitive development, and risk of infection (1). Microbial colonization of preterm (PT) infants born at ≤37 wks gestational age (GA) is affected by a complex interaction of clinical and iatrogenic factors, including exposure to prolonged rupture of membranes [rupture of membranes >18 h (PROM)], Cesarean-section (C-section) delivery, antibiotic exposure, and hospitalization (2). In addition, genetics influences microbiome composition, as demonstrated by greater similarity among PT multiplets than singletons (3).

In both term and PT infants, diet is one of the major drivers of the gut microbiota composition and function. Consumption of human milk (HM) vs. infant formula results in significant differences in the infant gut microbiota composition (4). Mother's own milk (MOM) is the preferred source of nutrition for PT infants. When MOM is unavailable, donor human milk (DHM) is recommended, followed by specialized formula for PT infants (5). Neither unfortified MOM or unfortified DHM are nutritionally sufficient to meet the nutritional needs of the PT infant (6) so they need fortification to achieve an adequate macronutrient and micronutrient content (7).

Previous studies have documented differences in the fecal microbiota of PT fed MOM or DHM vs. infant formula (2). Less evidence exists related to the potential differences in the microbiota of PT infants fed HM supplemented with a human milk-based fortifier (HMF) compared to a bovine milk-based fortifier (BMF). A prior study with a small sample size (<10 infants) did not identify significant differences between the gut microbiota of PT infants consuming HM with HMF or BMF (8). Furthermore, infants can transition from one type of feeding to another, thus, being exposed to HM, infant formula, and different types of fortifiers over the course of their hospitalization in the NICU. Thus, the goal of this study was to analyze the potential effect of different types of feeding (HM or PT formula) and type of fortifiers on the gut microbiota composition of PT infants, while considering other perinatal factors, the exposure to antibiotics and the effect of postnatal age. This study also sought to evaluate the impact of milk fortifiers on the growth velocity of hospitalized PT infants and associations with characteristics of the PT infant gut microbiota. We hypothesized that gut microbial characteristics of PT infants fed primarily HM and a HMF would significantly differ from those fed HM supplemented with a BMF or PT formula.

## Materials and Methods

### Study Participants

This cohort study was conducted between 2016 and 2018 at Carle Hospital in Urbana, IL. The study protocol was approved by the Institutional Review Board Committee of this same hospital (9, 10). Preterm infants were enrolled at birth and followed until discharge from the NICU. Participants were included if they were born <33 wks of gestation or <1,500 g at birth, and that were <72 h-old on admission to Carle Hospital. Infants were excluded if they had any major or lethal congenital anomalies or abdominal wall or intestinal defects. Informed consent was obtained from each infant's parent(s) or care provider(s) before study enrollment. Diagnoses of pregnancy complications, including clinical chorioamnionitis (bacterial infection of the membranes of the placenta and amniotic fluid) and PROM, were obtained from the mother's clinical chart. Additionally, maternal race, mode of delivery, GA, exposure to antibiotics, birth complications, head circumference, and weight (weekly) were retrieved from the medical records of each infant. Weight and head circumference *Z*-scores were calculated at birth and at discharge using the Fenton growth charts (11). Nursing staff from the NICU collected dietary information, including total daily feeding volume, volume of MOM, DHM, or PT formula, use of HMF (Prolact+H^2^MF® [Prolacta Bioscience, Industry, CA]) and use of BMF (Similac® human milk fortifier-hydrolyzed protein concentrated liquid [Abbott Nutrition, Columbus, OH]). The fortification protocol at Carle Hospital consists of exclusively HM (raw MOM or pasteurized DHM) supplemented with a fortifier according to a standardized fortification regimen. Human milk is fortified with HMF in infants born <28 GA, and after 32 wks corrected GA, BMF can be introduced. In infants born between 28 and 34 wks GA, HM is supplemented with BMF. After 34 wks corrected GA, infants can to PT formula if HM is not available. The weekly proportion of each specific type of milk was calculated and based on intake, infants were assigned as exclusively fed MOM, exclusively fed DHM, exclusively fed PT formula, or mixed fed. In the latter case, it was calculated what type of milk (MOM, DHM, or infant formula) they were predominantly fed. A freshly voided stool was collected from the infant's diaper by the nursing staff on a weekly or bi-weekly basis. After collection, samples were placed into coolers with dry ice, picked-up by research staff and stored at −80°C until further processing.

### DNA Extraction

Stool samples collected during the first days after birth are characterized by low bacterial DNA concentration (12). For this reason, bacterial DNA was extracted using two different commercial kits to optimize DNA yield. The Power Fecal DNA Isolation Kit (QIAGEN, Valencia, CA) was used for the first stool sample collected according to the manufacturer's instructions, with some modifications, including using a different bead beating tube, the mechanical lysis method, and the incubation temperature. Briefly, a total of 250 μg of stool (wet weight) were placed into a 2 mL Lysing Matrix E tube (MP Biomedicals. Santa Ana, CA). The stool sample and 600 μL of the C1 solution were then heated at 70°C for 10 min. Tubes were then shaken using the FastPrep-24 (MP Biomedicals. Santa Ana, CA) at 6.5 m/s for 45. The remaining steps were performed according to manufacturer's instructions. Bacterial DNA from the subsequent stool samples collected was extracted using the QIAamp Fast DNA Stool Mini Kit (QIAGEN, Valencia, CA), as previously described (3).

### Sequencing and Bioinformatics Analysis

The V3–V4 region of the 16S rRNA gene was amplified with the forward F357 (5′-CCTACGGGNGGCWGCAG-3′) and reverse R805 (5′- GACTACHVGGGTATCTAATCC-3′) primers. DNA was amplified through PCR reactions using the AccuPrime Taq DNA Polymerase System (Life Technologies, Carlsbad, CA) in a DNA Engine (Bio-Rad, Hercules, CA). Amplicons were mixed in equimolar concentration and submitted for sequencing at the Roy J. Carver Biotechnology Center at the University of Illinois Urbana-Champaign. Sequencing was performed using the Illumina MiSeq platform using v3 paired-reads (2 × 250 bp) (San Diego, CA). Sequencing files were processed using QIIME2 (13) version 2018.6. Samples were demultiplexed using the demux emp-paired plugin. Data was denoised by identifying and removing chimeras using the Divisive amplicon De-noising Algorithm 2 pipeline (14). Reads were trimmed at 17 and 21 bp in the forward and reverse reads, respectively. A phylogenetic tree of the samples was constructed using the QIIME2 recommended plugins. Operational Taxonomic Units (OTUs) were characterized to single nucleotide variants, and a representative sequence was used to taxonomically characterize the sequence using a Naïve Bayes classifier trained in the V3–V4 region against the Silva 132 database (15). Alpha diversity metrics (Observed OTUs and Shannon), and beta diversity metrics were calculated at an even sampling depth of 23,098 reads. Alpha diversity describes the species diversity withing a sample, while beta diversity measures the dissimilarity between communities (16).

### Statistical Analysis

Analyses were performed using RStudio V.1.3.1093. Taxonomic abundances (relative abundance) were transformed using the arcsine square root method. Linear mixed-effect models were used to estimate the effect of perinatal, anthropometric, and dietary factors on alpha diversity estimates. These variables were used to estimate their association with the taxonomic composition using Microbiome Multivariable Association Linear Models (MaASLin2) (9) with the Benjamin-Hochberg false discovery rate (FDR) correction. Perinatal, anthropometric, and dietary factors were included as fixed effects in the model, and each infant was included as random effects. Differences in beta diversity were calculated based on the Unweighted and Weighted UniFrac distances using permutational multivariable analysis of variance (Adonis) with 999 permutations using the vegan package (17).

Taxonomic composition at genus level was used to determine if any gut enterotypes existed in the PT infant gut across time. Analysis was performed as described by Arumugam et al. (18). First, sample clustering was done using the Jensen-Shannon divergence index and the Partitioning Around Medoids (PAM) syntax. Ideal number of clusters was determined using the Calinski-Harabasz index, this number was validated using the Silhouette Validation Technique. Following this, each stool sample was assigned to a specific enterotype. To evaluate the differential taxonomic abundance of each enterotype, Linear Discriminant Analysis (LDA) Effect Size (LefSe) (19) was conducted using the web-based Galaxy platform (http://huttenhower.sph.harvard.edu/galaxy).

Growth curves were calculated based on weight gain velocity (g/d) and corrected GA (GA at birth + postnatal age) using non-linear mixed-effects models from the nlme package (20). The intercept and slope were set as fixed effects, variance of the intercept and the slope as random effects, and the grouping parameter was each infant. Spearman correlation was used to assess the association between weight gain velocity and bacterial abundance.

## Results

A total of 102 PT infants were enrolled in the study. However, five infants died during the study and were excluded from the analysis; thus, the final analysis was done with a sample size of 97 PT infants. Table 1 shows the demographic characteristics of the included participants. According to the medical records, 18.6 and 15.5% of the infants were exposed to chorioamnionitis and PROM, respectively. A total of 62.9% were female, most infants were born *via* C-section (78.3%), and maternal race was predominantly white (73.2%). On average, infants were born at 28.9 ± 2.45 wks of gestation, weighed 1,273 ± 427 g, and had a head circumference of 26.3 ± 3.08 cm. A total of five infants (5.2%) had a birthweight *Z*-score below the 10th percentile, 12 infants (12.3%) fell below the 10th percentile for the head circumference *Z*-score. In the immediate postpartum period, 22.7% of participants were administered antibiotics, and 25.8% required mechanical ventilation. On average, the hospitalization period was 6.54 ± 3.10 wks (range: 1–16 wks). Infants were discharged at 35 ± 1.91 wks corrected GA, with a weight of 2,270 ± 556 g. At discharge, 17 (17.5%) and 24 (24.7%) infants fell below the 10th percentile for weight and head circumference, respectively. On average, 5 samples were collected per infant (range: 1–16).

**Table 1:** Characteristics of study participants.

**Characteristic**	**Birth** ***n* = 97**	**End of follow-up** ***n* = 97**
**Maternal race**, ***n*** **(%)**		
Black	26 (26.8)	–
White	71 (73.2)	–
**Chorioamnionitis**, ***n*** **(%)**	18 (18.6)	–
**PROM**, ***n*** **(%)**	15 (15.5)	–
**Infant sex**, ***n*** **(%)**		
Female	61 (62.9)	–
Male	36 (37.1)	–
**Mode of delivery**, ***n*** **(%)**		
Vaginal	21 (21.6)	–
C-section	76 (78.3)	–
**Gestational age, weeks**	29 (4)	–
Prematurity category^*^, ***n*** **(%)**		
Extremely PT	26 (26.8)	–
Very PT	56 (55.7)	–
Moderate to Late PT	17 (17.5)	–
**Postnatal age, weeks**	–	6 (4)
**Corrected GA, weeks**	–	35 (2)
**Weight, g**	1,242 (636)	2,327 (799)
**Weight** ***Z*****-score**	0.2 (1.26)	−0.49 (1.16)
**Weight** ***Z*****-score, percentile**	58 (46)	31 (37)
**Head circumference, cm**	33 (2.5)	33 (2.5)
**HC** ***Z*****-score**	0.07 (1.55)	−0.51 (1.24)
**HC** ***Z*****-score, percentile**	57.8 (46)	31 (42)
**Mechanical ventilation**, ***n*** **(%)**	25 (25.8)	0
**Use of antibiotics**, ***n*** **(%)**	22 (22.7)	4 (4.12)

*Data presented as n (%) for categorical data or median (IQR) for numerical data*.

* *Extremely PT: <28 wks GA; Very PT: 28–32 wks GA; Moderate to Late PT: 32–37 wks GA*.

*GA, gestational age; HC, head circumference; PROM, premature rupture of membranes; PT, preterm infant*.

### Feeding Characteristics

**Figure 1:**
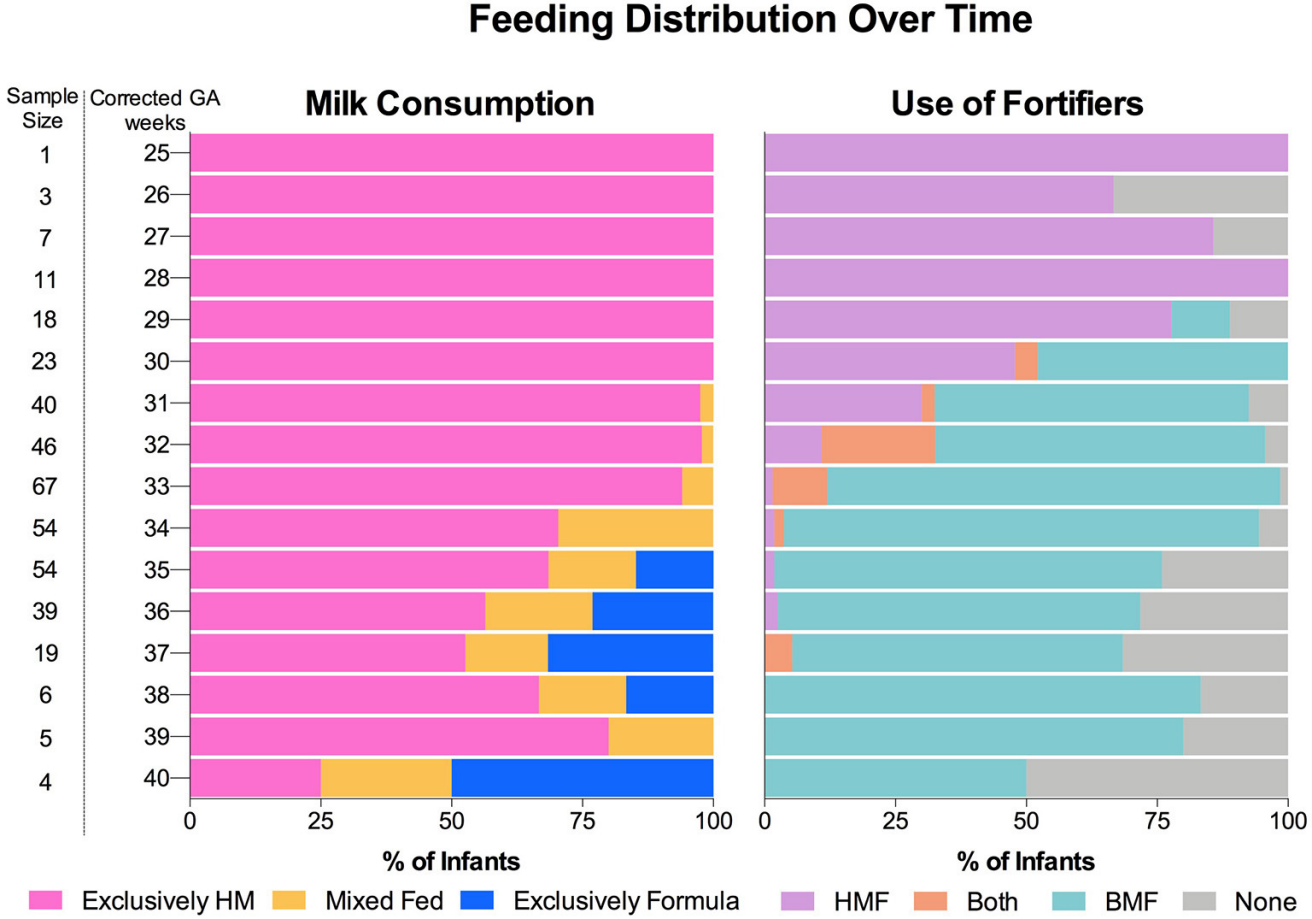
Feeding distribution of preterm infants during hospitalization period at the neonatal intensive care unit. Consumption of milk and use of fortifiers over time. Human milk and the use of a BMF was the most common regimen. HM, Human milk; HMF, human milk-based fortifier; BMF, bovine milk-based fortifier.

To evaluate the effect of the dietary exposures on the gut microbiota composition, feeding categories were created based on the type of milk consumed and the type of HM fortifier used. Donor human milk was provided when MOM was insufficient or not available. Only 17.5% infants (*n* = 17) were fed DHM at some point during their NICU stay (Supplementary Table 1). For this reason, DHM and MOM were combined into one group described as HM for all the analysis presented in this study. As shown in Figure 1, exclusively HM was consumed by the most PT infants (25 wks corrected GA) until 30 wks corrected GA. At this point, PT formula was introduced in combination with HM (mixed fed). Over time, the number of infants that were mixed fed, or fed exclusively PT formula increased. However, most of the infants were fed exclusively HM. The use of fortifiers followed a similar pattern. Human milk-based fortifier was fed from birth until a BMF fortification started at 30 wks corrected GA. Overall, PT infants, were fed the combination of both of fortifiers (HMF+BMF during most of the hospitalization period). Using the amount and type of feed (HM or formula) and the type of fortification (HMF or BMF), feeding categories were created to examine the effect of dietary factors on the gut microbiota composition of PT infants as shown in Table 2.

**Table 2:** Feeding categories.

**Category**	**Milk**	**Fortifier**
Human milk - none	Exclusively human milk or	None
	Mixed fed with >50% HM	
Human milk + HMF	Exclusively human milk or	HMF
	Mixed fed with >50% HM	
Human milk + BMF	Exclusively human milk or	BMF
	Mixed fed with >50% HM	
Human milk + both	Exclusively human milk or	HMF + BMF
	Mixed fed with >50% HM	
Formula + BMF	Exclusively formula or	BMF
	Mixed fed with >50% formula	
Formula	Exclusively formula or	None
	Mixed fed with >50% formula	

*HMF, human milk-based fortifier; BMF, bovine milk-based fortifier*.

### Early-Life Gut Microbiota Characteristics

Only 29 infants had a collected stool sample during the first week postpartum. Therefore, the first available stool sample was used to characterize the “early-life” gut microbiota of all study participants, which on average, was collected at 2.16 ± 1.13 wks postnatal age (31.1 ± 2.25 wks corrected GA). Analyses of alpha diversity revealed that PT infants from black mothers had significantly higher number observed OTUs than infants from white mothers (23.3 ± 10.3 vs. 17.5 ± 11). This difference remained significant after adjusting for corrected GA and exposure to antibiotics (Supplementary Figure 1A). Additionally, there was a linear relationship between corrected GA and observed OTUs (*p* = 0.02), when analysis was adjusted for infant's weight and exposure to antibiotics (Supplementary Figure 1B). No statistically significant associations were found between the number of observed OTUs and mode of delivery, exposure to chorioamnionitis, PROM, infant's sex, or mechanical ventilation (Supplementary Table 2). Similarly, there was no effect of any of these perinatal factors on the Shannon diversity index. Beta diversity, measured by Unweighted and Weighted UniFrac distances showed that exposure to PROM and infant's sex, each explained 2.3% of the variance of the Unweighted and Weighted UniFrac distances (*p* < 0.05). Maternal race explained 2.1% of the variance of the Weighted UniFrac distances (*p* = 0.021) (Supplementary Table 3). There were no other associations between beta diversity metrics and delivery mode, exposure to chorioamnionitis, use of mechanical ventilator and exposure to antibiotics.

Taxonomical analysis revealed significant differences in the early-life microbiota based on perinatal characteristics. There was a positive association between corrected GA and the abundance of *Enterococcus* and *Veillonella* (*p* = 0.0003, *q* = 0.01; *p* = 0.001, *q* = 0.018, respectively); analysis was performed adjusting for exposure to antibiotics (Supplementary Figure 2). Infants that were vaginally delivered had higher abundance of *Bacteroides* (8.76%) than infants born *via* C-section (1.63%); *p* = 0.05, *q* = 0.08. Infants that were exposed to chorioamnionitis during pregnancy had higher relative abundances of *Bifidobacterium* and *Enterobacter* (7.19 and 8.04%) compared to those that were not exposed (6.05 and 3.02%). PROM was significantly associated with higher abundances of *Bacteroides* (8.79 vs. 1.0%; *p* = 0.0001, *q* = 0.03). There were no statistically significant differences between infant's sex, maternal race, use of mechanical ventilation, infant's weight, and exposure to antibiotics with the early-life gut microbial composition.

### Changes in Gut Microbiota During the NICU Admission

**Figure 2:**
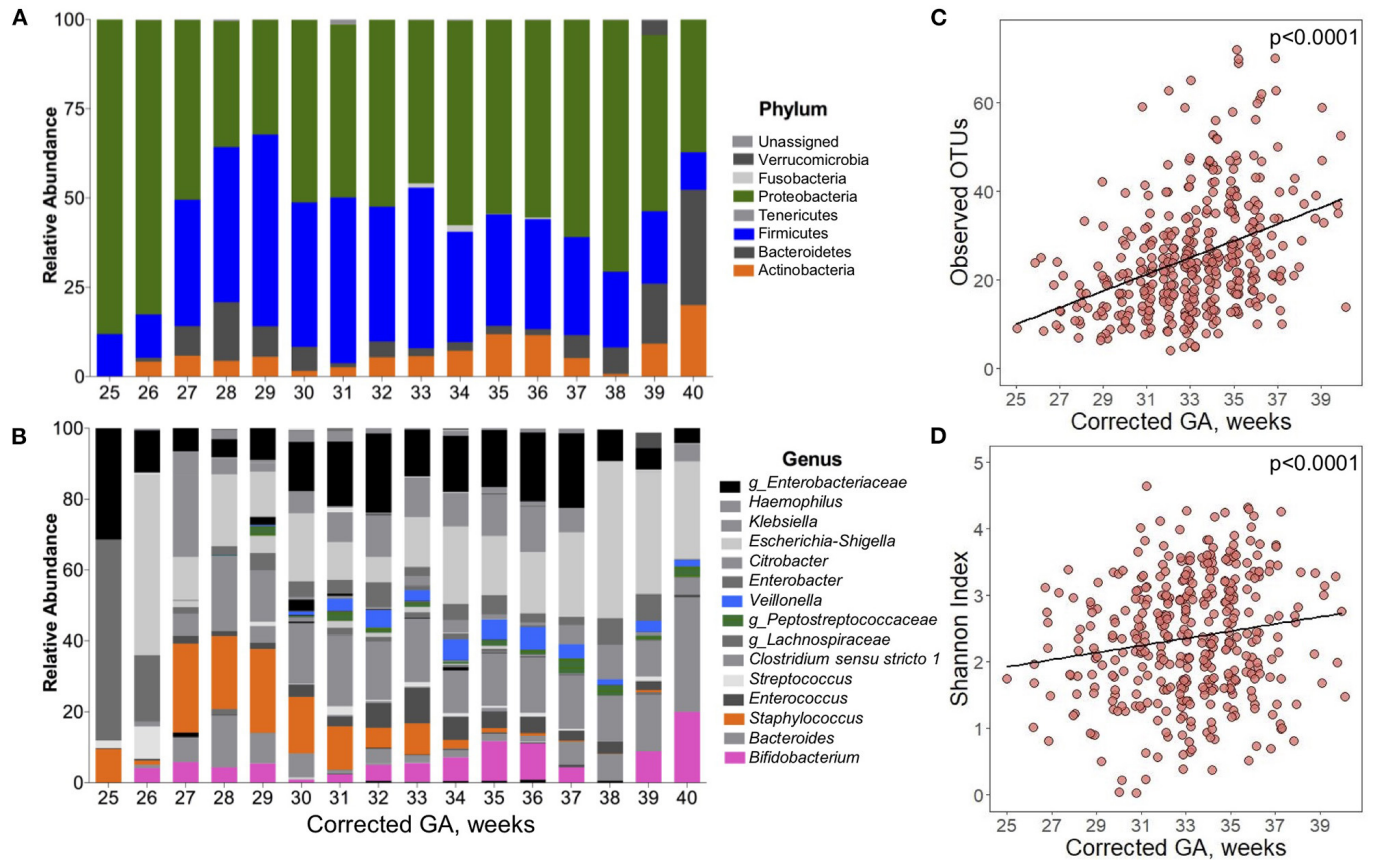
Postpartum development of the gut microbiota composition of preterm infants. Over time, there were significant changes in the composition of the gut microbiota of preterm infants. Taxonomical composition at **(A)** phylum and **(B)** genus level. Highlighted in color are the bacteria that significantly changed over time (*q* < 0.05). Differences calculated using Microbiome Multivariable Association Linear Models (MaASLin2) with infants as a random effect term. **(C)** Observed OTUs and **(D)** Shannon diversity Index significantly increased over time. Differences calculated using linear mixed-effect model with infants as a random effect term. Adjusted for antibiotic use. Corrected GA: gestational age at birth + postnatal age. GA, gestational age.

Over the course of their hospitalization, changes in gut microbial alpha diversity, beta diversity and taxonomic composition were observed. Significant changes in the abundance of the phyla Proteobacteria, Firmicutes, and Actinobacteria were found (Figure 2A). At the youngest ages (25–26 wks corrected GA), the gut microbiota was dominated by Proteobacteria with a mean relative abundance <85% ± 10.2 of total reads. After 27 wks corrected GA, the relative abundance of Proteobacteria started to decrease, and the relative abundance of Firmicutes increased. At corrected GA <27 wks, the relative abundance of Firmicutes was 12% ± 10.6 (25–26 wks corrected GA), and this increased over the NICU stay. Across time, the mean relative abundance of bacteria of the phylum Actinobacteria was 7% ± 13.2. At the genus level, statistically significant differences were found over time in the abundance of *Bifidobacterium, Staphylococcus*, uncharacterized *Peptostreptococcaceae*, and *Veillonella* (Figure 2B). In infants with <32 wks corrected GA, the average relative abundance of *Bifidobacterium* was 3.5% ± 6.7, which increased over time to 8.82% ± 12. The abundance of *Staphylococcus* had a significant decrease with greater corrected GA. From 25 to 30 wks corrected GA, the mean relative abundance of *Staphylococcus* was 16% ± 22, which was followed by a dramatic decrease in its abundance to 3% ± 1.27 by 34 wks corrected GA. The genera uncharacterized *Peptostreptococcaceae* and *Veillonella* reached a mean relative abundance >1% up to 29 and 31 wks corrected GA, respectively.

Time also had a significant effect on alpha diversity. There was a linear relationship between both Observed OTUs and Shannon Index with corrected GA, even after accounting for the use of antibiotics. On average, there was a 7.97 increment per week in the number of Observed OTUs (Figure 2C). Similarly, Shannon Index increased 0.095 units per week corrected GA (Figure 2D). During the hospitalization, multiple factors influenced beta diversity. Around 50% of the variation of the Unweighted and Weighted UniFrac distances was explained by each infant. Chronological variables (GA, postnatal age, and corrected GA) had a significant effect on beta diversity (Supplementary Table 4). Delivery mode explained 0.07 and 1.3% of the Unweighted and Weighted UniFrac distances, respectively. Exposure to chorioamnionitis had a significant effect on both Unweighted and Weighted UniFrac distances. Lastly, exposure to PROM, use of mechanical ventilation, and exposure to antibiotics had a significant effect only in Unweighted UniFrac distances.

**Figure 3:**
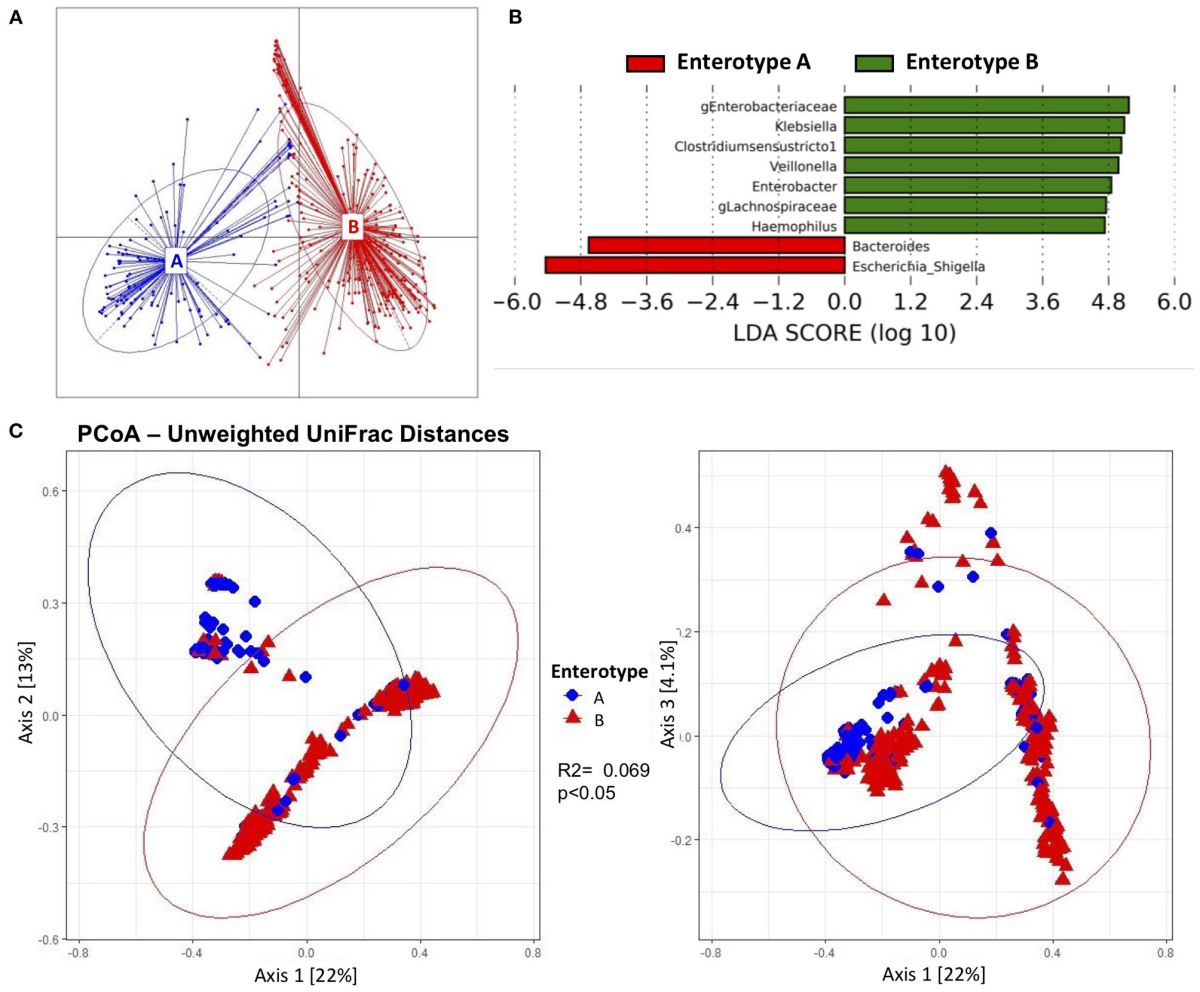
Beta diversity and enterotypes characteristics of gut microbiota from preterm infants. Beta diversity was significantly associated to two different enterotypes. **(A)** Two different enterotypes (Enterotype A and Enterotype B) were characterized. The majority of the samples belonged to Enterotype B. **(B)** Discriminative genera determined by linear discriminant analysis (LDA) showed bacteria that *Bacteroides* and *Escherichia-Shigella* were enriched in Enterotype A; whereas uncharacterized *Enterobacteriaceae, Klebsiella, Clostridium sensu stricto 1, Veillonella, Enterobacter*, uncharacterized *Lachnospiraceae* and *Haemopohilus* were enriched in Enterotype B. **(C)** Principal coordinate representation of Unweighted UniFrac Distances (Axis 1 vs. Axis 2, and Axis 1 vs. Axis 3) of the two enterotypes. Enterotypes explained 6.9% of the variance of the Unweighted UniFrac Distances.

When beta diversity was explored to assess the changes over time, the samples clustered into two distinct groups. For this reason, enterotype analysis was performed and two different enterotypes were found (Enterotype A and Enterotype B) (Figure 3A). Overall, 175 samples (32.8%) belonged to Enterotype A, whereas 358 samples (67.2%) were assigned to Enterotype B. To further understand the characteristics of the two clusters, LefSe analysis was performed. Enterotype A had enriched abundance of bacteria from the genus *Bacteroides* and *Escherichia-Shigella*. While bacteria from the genus uncharacterized *Enterobacteriaceae, Klebsiella, Clostridium sensu stricto 1, Veillonella, Enterobacter, g_Lachnospiraceae*, and *Haemophilus* were enriched in Enterotype B (Figure 3B). The most striking difference between the enterotypes was the relative abundance of *Escherichia-Shigella*; which was 45.4% in samples belonging to Enterotype A compared to 1.47% in samples belonging to Enterotype B (Supplementary Figure 3). Other genera that showed significant differences between the enterotypes were *Clostridium sensu stricto 1* (26.7% difference) and *g_Enterobacteriaceae* (25.9% difference). Furthermore, alpha diversity indices revealed that Enterotype B was more diverse than Enterotype A (Supplementary Figure 4). Samples from Enterotype B had 26.5 ± 13.1 Observed OTUs, and a Shannon diversity Index of 2.61 ± 0.78 compared to 23.2 ± 12.9 Observed OTUs and 1.9 ± 0.95 Shannon Index in samples belonging to Enterotype A which was statistically significant. The enterotype clustering explained 6.9% of the variance from the Unweighted UniFrac distances (*p* < 0.05) (Figure 3C), and 2.5% of the Weighted UniFrac distances; however, the latter was not statistically significant. Furthermore, when clinical characteristics were compared between the enterotypes, more samples from infants exposed to chorioamnionitis belonged to Enterotype B (25.7 vs. 17.1%). In Enterotype A, there was a significantly higher percentage of samples from infants consuming BMF (72 vs. 62%) (Table 3).

**Table 3:** Characteristics of stool samples belonging to each enterotype.

**Variable**	**Enterotype A** ***n* = 175 samples**	**Enterotype B** ***n* = 358 samples**	*p* ^*^
**Maternal race**, ***n*** **(%)**			
Black	32 (12.3)	81 (22.6)	0.26
White	143 (81.7)	277 (77.4)	
**Chorioamnionitis**, ***n*** **(%)**	30 (17.1)	92 (25.7)	0.028
**PROM**, ***n*** **(%)**	45 (25.7)	75 (20.9)	0.22
**Infant sex**, ***n*** **(%)**			
Female	114 (65.1)	229 (63.9)	0.84
Male	61 (34.8)	129 (36.0)	
**Mode of delivery**, ***n*** **(%)**			
Vaginal	46 (26.3)	87 (24.3)	0.67
C-section	129 (73.7)	271 (75.7)	
**Corrected GA, weeks**	33 (4)	33 (3)	0.90
**Weight, g**	1,758 (1,044)	1,744 (786)	0.94
**Use of antibiotics**, ***n*** **(%)**	16 (9.14)	27 (7.54)	0.51
**Milk consumed**, ***n*** **(%)**			
Human milk	164 (93.7)	322 (89.9)	0.19
Formula	11 (6.28)	36 (10.0)	
**Fortifier consumed**, ***n*** **(%)**			
None	16 (9.14)	49 (13.7)	0.15
HMF	24 (13.7)	68 (18.9)	0.14
BMF	126 (72.0)	222 (62.0)	0.025
Both	9 (5.14)	19 (5.31)	0.9
**Change enterotype**, ***n*** **(%)**	116 (66.3)	138 (38.5)	<0.001

*Data presented as n (%) for categorical data or median (IQR) for numerical data*.

*PROM, premature rupture of membranes; GA, gestational age; HMF, human milk-based fortifier; BMF, bovine milk-based fortifier*.

* *Differences based on Fisher's exact test for categorical variables or Kruskal-Wallis test for numerical variables*.

**Figure 4:**
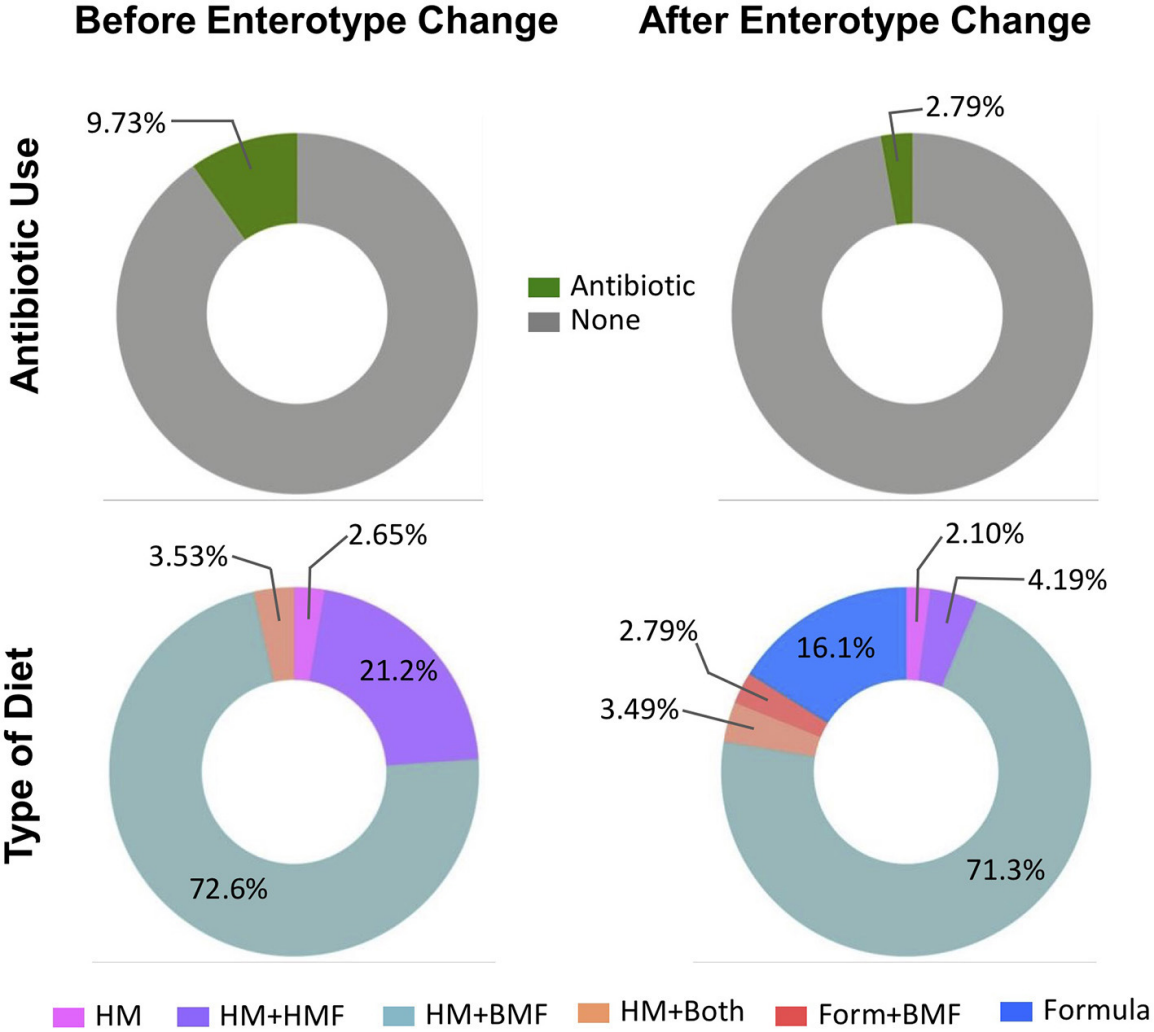
Characteristics before and after change in enterotype. Percentage of antibiotic use and diet category of infants prior moving from on enterotype to another. Before the change of the enterotype, there was a greater use of antibiotics, and HM in combination with fortifiers was the main diet category. Stool samples after they changed to another enterotype were associated with less antibiotic use, and with the introduction to formula feeding. HM, human milk; HMF, human milk-based fortifier; BMF, bovine milk-based fortifier; Form, formula.

From the total sample size (*n* = 97), 42 infants (43.3%) changed from one enterotype to another over the course of hospitalization. From the total number of samples belonging to Enterotype A, 116 samples (66.3%) moved to Enterotype B, compared to 138 samples (38.5%) that belonged to Enterotype B and moved to Enterotype A. Out of 97 studied infants, stool samples from 14 infants (14.4%) were associated exclusively to Enterotype A, samples from 41 infants (42.3%) exclusively to Enterotype B, and 42 infants (43.3%) had stool samples (254 stool samples) that changed from one enterotype to another. There were no statistically significant differences in the perinatal characteristics among infants exclusively belonging to one enterotype or infants that changed enterotypes (Supplementary Table 4). To further explore the possible variables associated to the change from one enterotype to another, chronological variables, use of antibiotics, and type of diet were assessed before and after samples changed to another enterotype. The mean corrected GA and body weight were significantly higher after enterotype change (31.7 wks corrected GA vs. 34.7 wks corrected GA and 1,578 g vs. 2,091 g, respectively; *p* < 0.05) (Supplementary Table 5). As shown in Figure 4, there was a higher percentage of antibiotics use prior to changing from one enterotype to another (9.73 vs. 2.79%, *p* = 0.037). There were significant associations in the dietary regimen and the change from one enterotype to another. Prior to the change to another enterotype, only HM with or without fortifiers was consumed. There was a higher percentage of HM+BMF use (72.6%), followed by HM+HMF (21.2%) and by the combination of both (HM+Both fortifiers: 3.53%) (Figure 4), there was no consumption of PT formula during this period. After changing to another enterotype, the use of infant formula increased to 16.1%. and HM+BMF remained as the most common feeding strategy.

**Figure 5:**
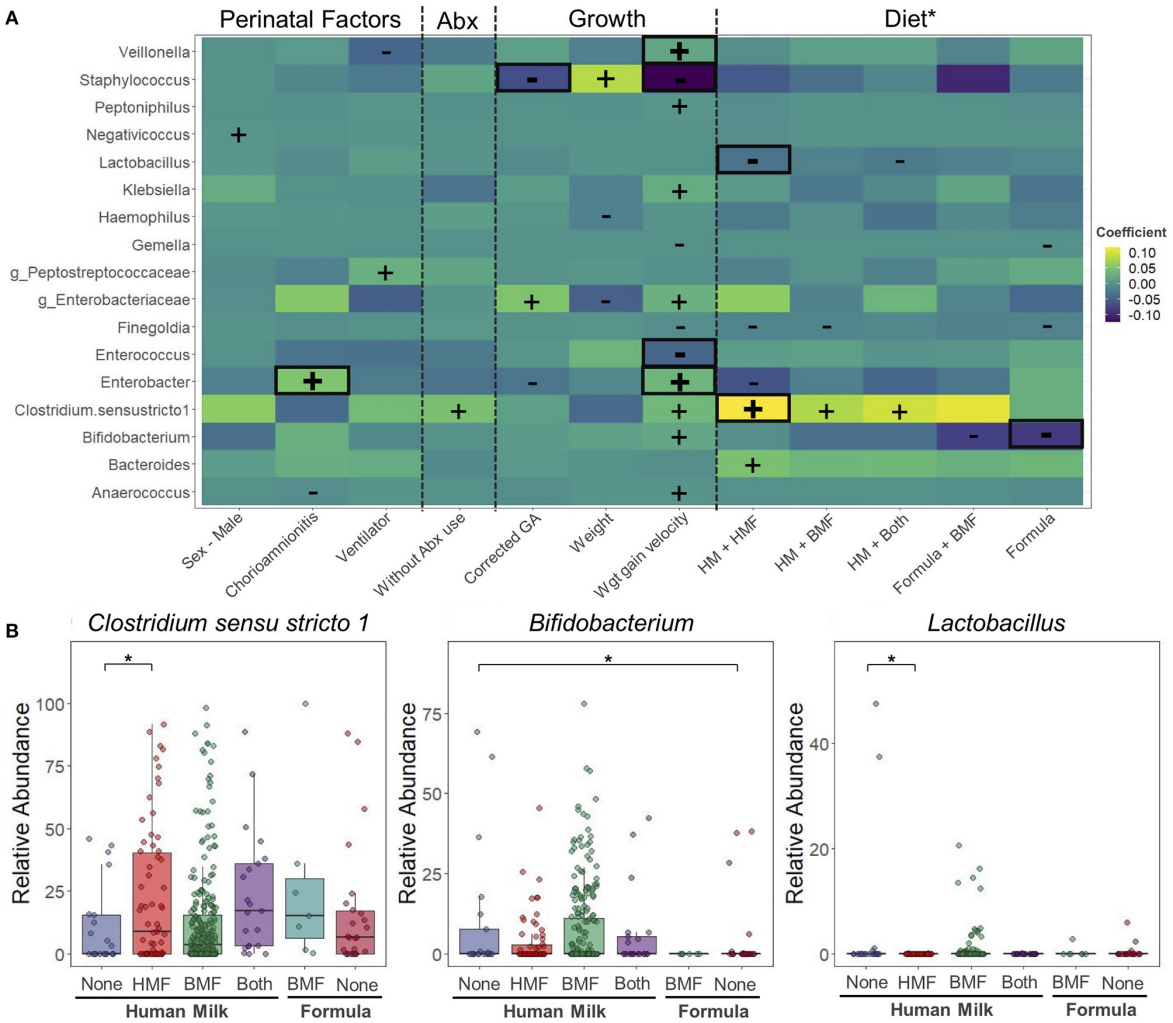
Changes in taxonomic composition during hospitalization period at the neonatal intensive care unit. Taxonomical changes associated to different perinatal factors. **(A)** Heatmap of associations between bacterial genera and perinatal factors, use of antibiotics, growth and diet. Positive (+) and negative (–) signs denote significant associations (*p* < 0.05). Highlighted in red are the associations that remained significant after false discovery rate adjustment (*q* < 0.05). Differences calculated using Microbiome Multivariable Association Linear Models (MaASLin2). Model included infant sex, exposure to chorioamnionitis, use of mechanical ventilation, exposure to antibiotics, corrected GA, body weight, weight gain velocity, and type of milk and fortifier consumed. Infant was included as a random effect term. **(B)** Bacteria significantly affected by dietary factors. *HM without fortification as control group. Corrected GA: gestational age at birth + postnatal age. Abx, antibiotic; PROM, premature rupture of membranes; GA, gestational age; HM, human milk; HMF, human milk-based fortifier; BMF, bovine milk-based fortifier; Wgt, Weight.

Another goal of this study was to evaluate the associations between perinatal factors, use of antibiotics, growth, and dietary exposures with the taxonomic composition over the course of the NICU stay. Multivariate analyses revealed that exposure to chorioamnionitis was associated with higher relative abundance of *Enterobacter* (7.07%) over time compared to infants never exposed to chorioamnionitis (3.86%). Other associations were found between perinatal factors and taxonomic composition, including a higher abundance of *Negativicoccus* in male PT infants, higher abundance of uncharacterized *Peptostreptococcaceae* with the use of mechanical ventilation, lower abundance of *Anaerococcus* when exposed to chorioamnionitis, and lower abundance of *Veillonella* when infants needed mechanical ventilation. During the hospitalization period, the only bacterium that seem to be affected by the use of antibiotics was *Clostridium sensu stricto 1*. However, these associations were no longer significant after FDR adjustment (*p* < 0.05 and *q* > 0.05) (Figure 5A). Growth variables [corrected GA, weight, and weight gain velocity (g/d)] were also analyzed in the multivariate analysis. Corrected GA and weight gain velocity were negatively associated with the abundance of *Staphylococcus* (*q* < 0.01). Another bacterium that was negatively associated with weight gain velocity was *Enterococcus* (*q* < 0.01). In contrast, there was a positive association between weight gain velocity and the abundance of *Veillonella* and *Enterobacter* (*q* < 0.01).

Dietary exposures were included in the multivariate analysis to evaluate their association with the taxonomic characteristics of study participants (Figure 5A). When different combinations of milk and milk fortifiers (HM-None, HM+HMF, HM+BMF, HM+Both, Formula+BMF, and Formula) were explored, 10 different associations with a *p* < 0.05 and *q* > 0.05 were found. Compared to HM consumption without any milk fortifier (HM-None), infants consuming HM+HMF had higher abundances of *Bacteroides*, and lower abundances of *Finegoldia* and *Enterobacter*. Infants consuming HM+BMF had higher abundance of *Clostridium sensu stricto 1*, and lower abundance of *Finegoldia*. When infants were exposed to HM with the combination of both fortifiers (HM+Both) they showed higher abundances of *Clostridium sensu stricto 1*, and lower abundances of *Lactobacillus* when comparing to HM-None. The consumption of PT formula alone or with BMF decreased the abundance of *Gemella, Finegoldia*, and *Bifidobacterium*. Overall, only three associations remained significant (*p* < 0.05 and *q* < 0.05) (Figure 5B). Compared to infants consuming HM-None, infants consuming HM with a HMF had higher abundances of *Clostridium sensu stricto 1* (HM-None: 10.8%; HM-HMF: 22.2%) and lower abundance of *Lactobacillus* (HM-None: 4.13%; HM-HMF <0.1%). Compared to consuming HM-None, infants consuming formula had significantly lower levels of *Bifidobacterium* (HM-None: 9.82%; Formula: 4.28%).

### Effect of Milk Fortifiers on Growth Rates

**Figure 6:**
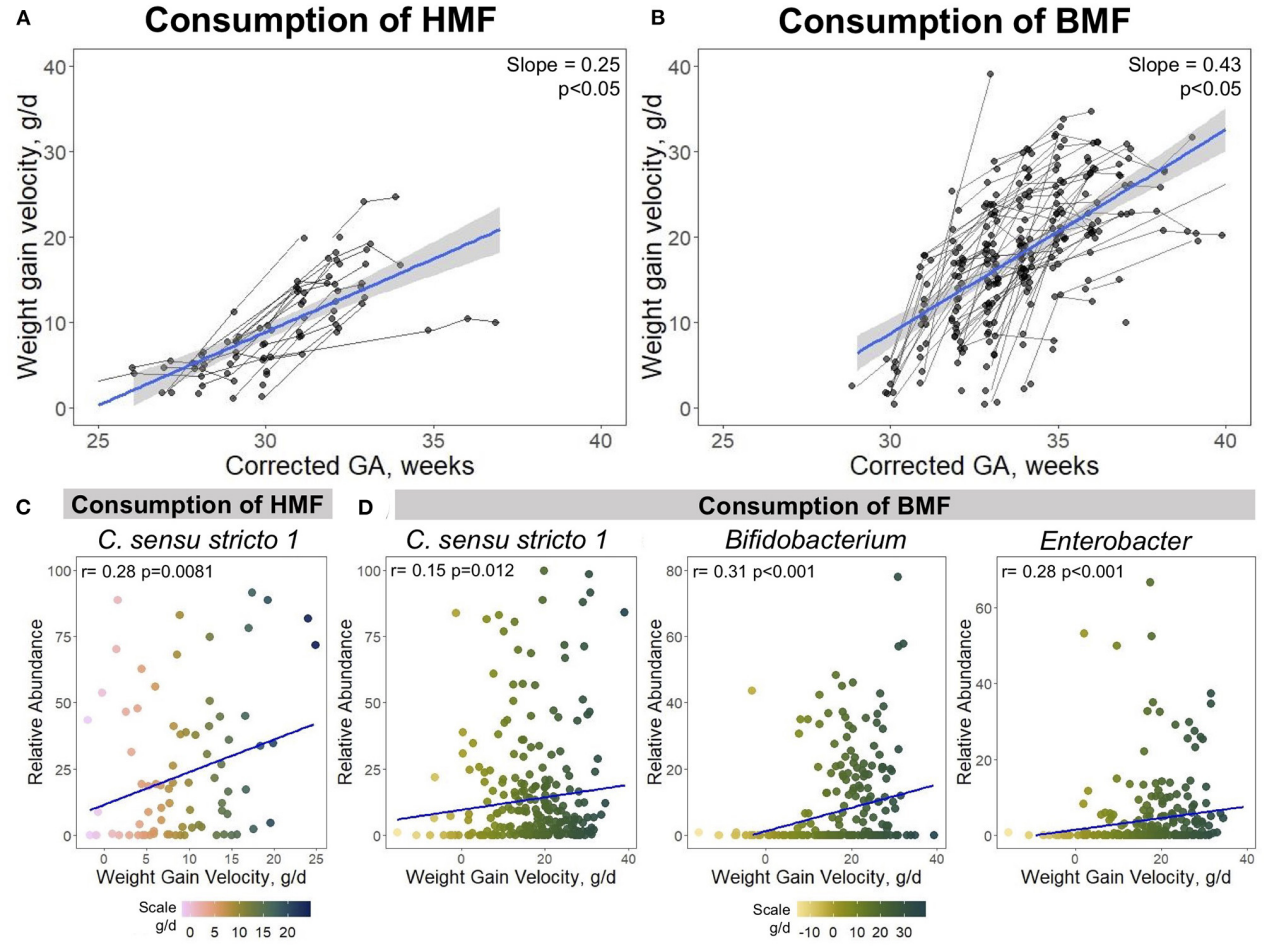
Associations between growth velocity, use of fortifiers and taxonomic composition. The use of HMF and BMF are associated with the infant's growth and their taxonomic composition. **(A)** Growth curves of PT infants when consuming HMF, or **(B)** BMF. Spearman correlations showing positive associations between *Clostridium sensu stricto 1, Bifidobacterium*, and *Enterobacter* with weight gain velocity when infants were exposed to **(C)** HMF or **(D)** BMF. Corrected GA: gestational age at birth + postnatal age. GA, gestational age; HMF, human milk-based fortifier; BMF, bovine milk-based fortifier.

A goal of HM fortification is to increase the macro and micronutrient content to ensure appropriate growth in PT infants. The HMF was used in PT infants <29 wks corrected GA. BMF was initiated around 29 weeks corrected GA and was the most commonly used fortification strategy in this study. Both fortifiers had a significant effect on the growth rate, measured by weight gain velocity (g/d). The overall growth rate in the study participants was 15 ± 9.76 g/d. This rate had a 0.39 increment per week, which was lower (0.25 g/d) when infants consumed a HMF (Figure 6A), and it was higher (0.43 g/d) when BMF was used (Figure 6B).

Next, associations between dietary intake, growth and microbial taxa were investigated. As previously mentioned, bacteria that were significantly associated with growth and/or dietary factors were *Clostridium sensu stricto 1, Bifidobacterium*, and *Enterobacter* (Figure 5A). Correlation analysis was performed between these bacteria abundances and weight gain velocity when infants were consuming either a HMF or BMF. There was a significant association between the abundance of *Clostridium sensu stricto 1* and weight gain velocity when fortification with a HMF was used (rho = 0.28, *p* = 0.0081) (Figure 6C). During the fortification with BMF, positive correlations were found between weight gain velocity and the abundance of *Clostridium sensu stricto 1* (rho = 0.15, *p* = 0.012), *Bifidobacterium* (rho = 0.31, *p* < 0.001), and *Enterobacter* (rho = 0.28, *p* < 0.001) (Figure 6D). There were no significant correlations between *Bifidobacterium* or *Enterobacter* abundance and weight gain velocity during fortification with HMF.

## Discussion

Differences in gut microbial structure associated with different perinatal and dietary characteristics were identified in this study. In the first collected stool sample after birth, the gut microbiota of infants of black mothers had a more diverse gut microbiota, which was significantly associated with beta diversity metrics. This could be due to characteristics of the maternal vaginal and cervical microbiota during pregnancy. The vertical transmission of microbes has been previously described (21). Mitchell et al. reported that seeding of maternal microbiota to the newborn's gut can be attributed to mother's rectal microbiota and not from the vaginal microbiota (22). However, PT infants are more likely to be born *via* C-section, a trend (*q* > 0.05) also observed in this study. While it remains controversial, some studies have shown that the womb is not a sterile environment, and the exposure to maternal microbes might initiate during pregnancy (23). Previous studies have shown that the vaginal microbiota of African American and Hispanic mothers had significantly higher alpha diversity compared to Asian and Caucasian mothers (24). Non-Hispanic black pregnant women have a more diverse cervical microbiota than pregnant white women (25). This effect of maternal race on infant gut microbial composition should be further explored.

Pregnancy complications were significantly associated with the gut microbiota of PT infants. Exposure to PROM significantly increased the abundance of *Bacteroides*, which could be associated with the maternal vaginal microbiota. There is a higher risk of PROM in pregnant women with bacterial vaginosis (26), which is characterized by lower *Lactobacillus* abundance and a bloom of other bacteria, including *Bacteroides* (27, 28). In the present study, PT infants exposed to chorioamnionitis had higher abundances of *Bifidobacterium* and *Enterobacter*, and the association between chorioamnionitis and *Enterobacter* abundance remained significant over the duration of the study. *Enterobacter* is a Gram-negative bacterium, which is a common early colonizer of the PT gut (29). Chorioamnionitis is an infection of the placenta membranes and the amniotic fluid. Evidence has suggested that there is an association between chorioamnionitis and the presence of *Enterobacteriaceae* (30), particularly of *Enterobacter* species (31) in placental tissues. In comparison to PROM, chorioamnionitis had a longer effect on the gut microbiota of PT infants, since it continued to have an association throughout the duration of hospitalization in the NICU.

After delivery, PT infants face a number of medical conditions secondary to prematurity, such as respiratory distress syndrome due to surfactant deficiency in the lungs. The most common treatment for this condition is mechanical ventilation. Bacteria of the genus *Veillonella* tended to be lower in infants exposed to respiratory support. *Veillonella*, an anaerobic bacterium, is a common colonizer of the newborn gut (10). This bacterium utilizes lactate from carbohydrate fermentation (10) and is able to produce propionate (32), which could be beneficial for the host. Another common medical treatment in PT infants is the use of antibiotics immediately after birth and during the hospitalization. Thus, we investigated the effect of antibiotics on the PT gut. Although it failed to remain significant after FDR correction, infants not exposed to antibiotics had higher abundances of *Clostridium sensu stricto 1*, which suggests the effect of antibiotics on PT gut microbiome. Members from the cluster of *Clostridium sensu stricto 1* have been associated with prematurity and increased incidence of necrotizing enterocolitis (33). Although the associations mentioned above failed to remain significant, it is important to point out these differences so further studies can be conducted looking at these factors.

Chronological and anthropometric variables such as corrected GA and body weight, are also associated with PT gut colonization. Herein, a positive association was observed between diversity metrics, taxonomic composition and corrected GA. Relative abundance of *Staphylococcus*, a facultative anaerobe, significantly decreased over the course of hospitalization, which is a common feature reported in PT infants (34–36). Higher abundance of *Staphylococcus* has been associated with C-section delivery (37), breastfeeding, and HM feeding (38, 39), suggesting that the source can be the mother's skin and/or the milk microbiota. The decrease in the relative abundance of *Staphylococcus* coincided with an increase abundance of other common colonizers of the newborn gut, including *Veillonella* and *Bifidobacterium*. In PT infants, there is a delayed colonization with *Bifidobacterium* (40, 41), but this increases over time (35, 42–44). The concurrent increase of *Veillonella* and *Bifidobacterium* could be attributed to their mutualistic relationship. As mentioned above, *Veillonella* metabolizes lactate for propionate production. *Bifidobacterium* is a strict anaerobe capable of digesting complex carbohydrates, such as human milk oligosaccharides (HMOs) and producing acetate, formate (45) and lactate (46), which then is utilized by *Veillonella*.

Enterotype analysis revealed that stool samples from PT infants clustered into two distinct groups (Enterotype A and Enterotype B). Enterotype A was characterized by a lower diversity, and enrichment of *Bacteroides* and *Escherichia*-*Shigella*. In contrast, stool samples in Enterotype B had greater diversity and higher abundances of members from the phyla Proteobacteria (*Enterobacteriaceae*/*Enterobacter, Haemophilus*, and *Klebsiella*) and Firmicutes (*Clostridium sensu stricto 1, g_Lachnospiraceae*, and *Veillonella*). Enterotypes were first characterized by Arumugam and coworkers in 2011, by describing three fecal enterotypes in adult populations: *Bacteroides*-enriched, *Prevotella*-enriched, and *Ruminococcus*-enriched, which were strongly associated to the dietary habits (18). In pediatric populations, authors have described the existence of enterotypes. Zhong et al. analyzed the gut microbiota of school-age children and found three enterotypes: *Bacteroides*-dominated, *Prevotella*-dominated, and *Bifidobacterium*-dominated (47). A recent study reported that the gut microbiome of infants aged 0–6 months was characterized by three enterotypes: *Bifidobacteriaceae*-dominated, *Enterobacteriaceae*-dominated, and *Bacteroidaceae*-dominated (48). The enterotype enriched in *Bifidobacteriaceae* was characterized by an increased capacity of HMO utilization (48). In a similar analysis, Tauchi et al. (41) analyzed stool samples of PT and full-term infants using Weighted UniFrac distances to characterize the gut microbiota composition. These authors found three different clusters: *Bifidobacteriaceae*-dominated, *Enterobacteriaceae*-dominated and Gram-positive bacilli cocci (*Staphylococcaceae, Streptococcaceae*, or *Enterococcaceae*)-dominated. However, they reported that no cluster that was significantly associated exclusively to PT infants (41). Compared to adults, the gut microbiota of newborns is highly variable, changes drastically during the first weeks after birth, and could be affected by the environment the infant is developing (49). This could explain the variability and the lack of consistency in studies analyzing enterotypes in a pediatric population.

In this study, around 40% of the stool samples transitioned from one enterotype to another. The main factors associated with this transition were corrected GA, body weight, use of antibiotics and dietary characteristics. About 70% of the samples transitioned from Enterotype A (*Bacteroides* and *Escherichia-Shigella*-dominated) to Enterotype B (Proteobacteria and Firmicutes-dominated). Prior to the change from one enterotype to another, infants were receiving more antibiotics, and consuming primarily HM plus fortification (HMF and/or BMF), and there was no exposure to PT formula. After the transition to another enterotype, there was less exposure to antibiotics and the consumption of PT formula increased. The use of antibiotics in PT infants has been associated with higher abundances of *Bacteroides* (50) and *Escherichia-Shigella* (51). Additionally, Ma et al. reported that in full-term infants, *Bacteroides* abundance was significantly higher in breast-fed compared to formula-fed infants (52). Bacteria enriched in Enterotype B (*Enterobacteriaceae, Klebsiella, Clostridium sensu stricto 1, Veillonella, Enterobacter*, uncharacterized *Lachnospiraceae*, and *Haemophilus*) have been previously associated to the gut microbiota of PT infants. Evidence has shown that *Enterobacteriaceae/Enterobacter* increase with postnatal age in PT infants (41, 44, 53–55). Moreover, two different studies have suggested that the gut microbiota composition of PT infants, transitions over time from a *Staphylococcus* dominated (56) or Gram-positive bacilli cocci dominated (41), to an *Enterobacteriaceae*/*Enterobacter* dominated. Abundances of *Klebsiella* (55), *Clostridium* (57) and *Veillonella* (34, 57) have been found to increase with postnatal age in the gut of PT infants. Changes from one enterotype to another might not be attributed to a specific factor (e.g., greater use of infant formula). Instead, it might be the combination of multiple characteristics (clinical and dietary) contributing to the modification of the gut microbiota. The effect of the feeding type (human milk, infant formula, and type of fortifiers) and their association with the gut microbiota of PT infants is understudied. Furthermore, studies using clustering techniques such as enterotypes to assess the gut microbiota in a PT population are scarce. Hence, there is insufficient evidence on the clinical relevance of PT infants belonging or transitioning from one enterotype to another. Therefore, more research is needed to fully understand the implications of the gut microbiota clustering and feeding types in PT infants.

This study also explored the associations between taxonomic composition and dietary distribution over the course of hospitalization in the NICU. When perinatal and chronological indicators, as well as antibiotic exposure were considered, three different bacteria were significantly associated with the type of milk and/or fortifier consumed: *Clostridium sensu stricto 1, Bifidobacterium*, and *Lactobacillus*. Compared to HM alone, the consumption of HM with a HMF was associated with higher abundances of *Clostridium sensu stricto 1* and lower abundances of *Lactobacillus*, and consumption of infant formula was associated with lower abundances of *Bifidobacterium*. Evidence about the effect of milk fortifiers on the gut microbiota composition is scarce. Underwood and co-workers conducted a longitudinal study evaluating the effect of a HMF or BMF added to HM in the PT gut microbial composition. Over time, the abundance of the genus *Lactobacillus* decreased with the use of HMF (8). Additionally, prior studies have suggested that HM consumption (MOM or DHM) is associated with higher abundances of *Clostridium* in PT infants (58, 59). However, these studies consisted of only one time point analysis (59), or had small sample sizes (8, 58). In PT infants, the effect of HM consumption on *Lactobacillus* needs further exploration. Studies have reported both increased (59) and decreased (60) abundance of this microbe with the consumption of HM compared to specialized PT infant formula. *Lactobacillus* is a common colonizer of the gut microbiota capable of metabolizing carbohydrates to produce lactic acid (61). Supplementation of members of *Lactobacillus* to PT infants is associated with a reduction in the abundance of *Clostridium histolyticum* (62), and with an increase in beneficial bacteria such as *Bifidobacterium* (63) compared to those infants not receiving probiotic supplementation. The observed difference in fecal *Bifidobacterium* abundance between infants fed HM vs. PT formula in this study is consistent with the published literature in preterm (44, 59) and full-term infants (64).

Lastly, this study aimed to identify possible associations between the use of milk fortifiers and grow indicators. Weight gain velocity, commonly expressed as g/d, is used to estimate growth and development of PT infants. In the present study, over the course of hospitalization there was a linear growth trajectory. However, when infants were consuming a BMF, weight gain velocity was significantly faster compared to when infants were consuming HMF (5). The use of milk fortifiers (HMF or BMF) are recommended in PT infants fed HM due to the insufficient macro- (particularly protein) and micronutrient (particularly calcium and phosphate) content of HM compared to the PT infant's growth requirements (7). The amount of fortification with either of these two fortifiers depends on the infant's requirements, and similar protein concentration can be achieved either of both fortifiers (65). Thus, differences in growth trajectories might be associated to the types of protein in HM and bovine milk. Zhang and collaborators demonstrated that there are around 33 proteins associated with the milk fat globule membrane, and 16 whey proteins, that are significantly different between HM and bovine milk (66). On the other hand, PT birth is often associated with restricted growth (67). After birth, around 90% of infants with restricted growth exhibit a catch-up growth trajectory, during which, weight gain increases to the expected growth curve (68). However, a meta-analysis conducted by Martin and collaborators analyzing the health impacts of catch-up growth trajectory in low-birth weight infants suggested that while rapid growth might have beneficial outcomes in the short-term, there might be adverse long-term outcomes associated with catch-up growth (69). This suggests that future studies should assess the weight gain velocities of low-birth-weight infants, particularly when fed HMF or BMF. Evidence of the association between growth trajectories and the gut microbial composition in infants is limited. A recent study revealed that gut microbial diversity and taxonomic characteristics are associated with the growth trajectories from 1 to 5 years of age (70). Prospective studies assessing the relationships between diet, growth and gut microbiota could serve to develop biomarkers to predict the possible infant's growth characteristics.

This study longitudinally analyzed the development of the gut microbiota of PT infants and compared different feeding types and milk fortifiers, however, there are some potential limitations worth mentioning. First, a pregnancy complication included in this study and significantly associated with the gut microbiota composition was chorioamnionitis. However, this affection was clinically diagnosed and not confirmed by culture or histologic tests. Another limitation in this study is that the only information available from the infant's mother was race. Additional data related to maternal health status could help find possible links between the infant's gut microbiota and maternal characteristics. Next, multiple stool samples were collected during the time infants were hospitalized in the NICU; however, the time point and the time interval when the stool samples were collected was not consistent across participants. In the same manner, another limitation is that the gut microbiota characteristics after birth were evaluated based on the first stool sample available, which was about 2 weeks after birth. Moreover, given the nature of the study, the results presented here might be biased toward associations mostly related to infants very-low birth weight infants (smaller GA and lower birth weight) which are more likely to be exposed to different milk fortifiers (HMF and BMF) to have longer hospitalization periods and a larger number of stool samples collected. Another limitation is the sample size. Albeit having a sample size of 97 PT infants with multiple sample collection, gut microbiome studies analyzing a large number of variables related to diversity and taxonomic composition could be underpowered. This could explain why some associations failed to remain significant after FDR correction. Lastly, we didn't account for the effects of the environment surrounding infants hospitalized in the NICU that could affect their gut microbiome (2). These factors include transition from incubators to open cribs and skin-to-skin care. Therefore, future longitudinal studies should aim to characterize the gut microbiota of PT infants based on their feeding type with larger sample sizes and considering the effect of other environmental aspects.

## Conclusion

This study found that PT infants from Black or Hispanic mothers have a distinct gut microbiota soon after birth, but these differences do not persist over time. Pregnancy complications, particularly chorioamnionitis, can have a long-term effect on the fecal taxonomic composition. The use of HM with a HMF, and infant formula consumption significantly modified the abundances of *Clostridium sensu stricto 1* and *Bifidobacterium*. The use of HMF or BMF contributes to weight gain velocity, the latter associated with a more rapid weight gain. Also, these fortifiers were positively correlated with the abundance of *Clostridium sensu stricto 1, Bifidobacterium* and *Enterobacter*. Lastly, two different enterotypes associated with PT infants were characterized. The difference between these enterotypes was mainly in diversity metrics, and enrichment of *Bacteroides, Escherichia-Shigella*, uncharacterized *Enterobacteriaceae, Klebsiella, Clostridium sensu stricto 1, Veillonella, Enterobacter*, uncharacterized *Lachnospiraceae* and *Haemophilus*. A significant proportion of infants changed from one enterotype to another, which was associated with corrected GA, body weight, administration of antibiotics and consumption of different HM or infant formula and different types of fortifiers. More studies are needed to further explore the effect of not only HM or formula but also HMF vs. BMF on the gut microbiota composition of PT infant.

## Data Availability Statement

The datasets presented in this study can be found in online repositories. The names of the repository/repositories and accession number(s) can be found at: https://www.ncbi.nlm.nih.gov/, PRJNA734298.

## Ethics Statement

The studies involving human participants were reviewed and approved by Institutional Review Board, Carle Hospital. Written informed consent to participate in this study was provided by the participants' legal guardian/next of kin.

## Author Contributions

TH, CW, and SD conceived the study. TH and SD designed the study. TH, CW, and AM collected samples analyzed in the study. CW and AM collected the clinical data for this study. MA-L analyzed the fecal microbiome and conducted bioinformatic and statistical analyses. MA-L, TH, CW, and SD wrote the manuscript. All authors approved the final manuscript prior publication.

## Funding

This study was funded by Carle-Illinois Collaborative Research Seed Proposal Program (TH and SD). MA-L was supported by a fellowship from Consejo Nacional de Ciencia y Tecnologia (CONACyT).

## Conflict of Interest

The authors declare that the research was conducted in the absence of any commercial or financial relationships that could be construed as a potential conflict of interest.

## Publisher's Note

All claims expressed in this article are solely those of the authors and do not necessarily represent those of their affiliated organizations, or those of the publisher, the editors and the reviewers. Any product that may be evaluated in this article, or claim that may be made by its manufacturer, is not guaranteed or endorsed by the publisher.

## Abbreviations

BMF: bovine milk-based fortifier
GA: gestational age
HM: human milk
HMF: human milk-based fortifier
HMOs: human milk oligosaccharides
LEFSE: linear discriminant analysis effect size
MaASLin2: microbiome multivariate association linear models
NICU: neonatal intensive care unit
OTU: operational taxonomic unit
PROM: premature rupture of membranes
PT: preterm.

## References

[1] Robertson RC Manges AR Finlay BB Prendergast AJ. The human microbiome and child growth – first 1000 days and beyond. Trends Microbiol. (2019) 27:131–47. 10.1016/j.tim.2018.09.008 30529020

[2] Aguilar-Lopez M Dinsmoor AM Ho TTB Donovan SM. A systematic review of the factors influencing microbial colonization of the preterm infant gut. Gut Microbes. (2021) 13:1–33. 10.1080/19490976.2021.1884514 33818293 PMC8023245

[3] Lim SJ Aguilar-Lopez M Wetzel C Dutra SVO Bray V Groer MW. The effects of genetic relatedness on the preterm infant gut microbiota. Microorganisms. (2021) 9:278. 10.3390/microorganisms9020278 33572789 PMC7911719

[4] Ho NT Li F Lee-Sarwar KA Tun HM Brown BP Pannaraj PS. Meta-analysis of effects of exclusive breastfeeding on infant gut microbiota across populations. Nat Commun. (2018) 9:4169. 10.1038/s41467-018-06473-x 30301893 PMC6177445

[5] Quigley M Embleton ND McGuire W. Formula versus donor breast milk for feeding preterm or low birth weight infants. Cochrane Database Syst Rev. (2019) 7:CD002971. 10.1002/14651858.CD002971.pub5 31322731 PMC6640412

[6] Hay WW Thureen P. Protein for preterm infants: how much is needed? How much is enough? How much is too much? Pediatr Neonatol. (2010) 51:198–207. 10.1016/S1875-9572(10)60039-3 20713283

[7] Arslanoglu S Boquien CY King C Lamireau D Tonetto P Barnett D. Fortification of human milk for preterm infants: update and recommendations of the European Milk Bank Association (EMBA) working group on human milk fortification. Front Pediatr. (2019) 7:76. 10.3389/fped.2019.00076 30968003 PMC6439523

[8] Underwood MA Kalanetra KM Bokulich NA Mirmiran M Barile D Tancredi DJ. Prebiotic oligosaccharides in premature infants. J Pediatr Gastroenterol Nutr. (2014) 58:352–60. 10.1097/MPG.0000000000000211 24135979

[9] Mallick H Rahnavard A McIver LJ Ma S Zhang Y Nguyen LH. Multivariable association discovery in population-scale meta-omics studies. bioRxiv [Preprint]. (2021). 10.1101/2021.01.20.427420 34784344 PMC8714082

[10] Milani C Duranti S Bottacini F Casey E Turroni F Mahony J. The first microbial colonizers of the human gut: composition, activities, and health implications of the infant gut microbiota. Microbiol Mol Biol Rev. (2017) 81:e00036–17. 10.1128/MMBR.00036-17 29118049 PMC5706746

[11] Fenton TR. A new growth chart for preterm babies: Babson and Benda's chart updated with recent data and a new format. BMC Pediatr. (2003) 3:13. 10.1186/1471-2431-3-13 14678563 PMC324406

[12] Kalyuzhanaya MG Waite DW Roose-Amsaleg CL Payne MS Stinson LF Keelan JA. Comparison of meconium DNA extraction methods for use in microbiome studies. Front Microbiol. (2018) 9:270. 10.3389/fmicb.2018.00270 29515550 PMC5826226

[13] Bolyen E Rideout JR Dillon MR Bokulich NA Abnet CC Al-Ghalith GA. Reproducible, interactive, scalable and extensible microbiome data science using QIIME 2. Nat Biotechnol. (2019) 37:852–7. 10.1038/s41587-019-0209-9 31341288 PMC7015180

[14] Allali I Arnold JW Roach J Cadenas MB Butz N Hassan HM. A comparison of sequencing platforms and bioinformatics pipelines for compositional analysis of the gut microbiome. BMC Microbiol. (2017) 17:194. 10.1186/s12866-017-1101-8 28903732 PMC5598039

[15] Quast C Pruesse E Yilmaz P Gerken J Schweer T Yarza P. The SILVA ribosomal RNA gene database project: improved data processing and web-based tools. Nucleic Acids Res. (2013) 41:D590–6. 10.1093/nar/gks1219 23193283 PMC3531112

[16] Jurasinski G Retzer V Beierkuhnlein C. Inventory, differentiation, and proportional diversity: a consistent terminology for quantifying species diversity. Oecologia. (2009) 159:15–26. 10.1007/s00442-008-1190-z 18953572

[17] Jari Oksanen F Blanchet G Friendly M Kindt R Legendre P McGlinn D. vegan: Community Ecology Package. (2020). Available online at: https://cran.r-project.org/package=vegan

[18] Arumugam M Raes J Pelletier E Le Paslier D Yamada T Mende DR. Enterotypes of the human gut microbiome. Nature. (2011) 473:174–80. 10.1038/nature09944 21508958 PMC3728647

[19] Segata N Izard J Waldron L Gevers D Miropolsky L Garrett WS. Metagenomic biomarker discovery and explanation. Genome Biol. (2011) 12:R60. 10.1186/gb-2011-12-6-r60 21702898 PMC3218848

[20] Pinheiro J Bates D DebRoy S Sarkar D RCT. nlme: Linear and Nonlinear Mixed Effects Modelse. (2021). Available online at: https://cran.r-project.org/package=nlme

[21] Asnicar F Manara S Zolfo M Truong DT Scholz M Armanini F. Studying vertical microbiome transmission from mothers to infants by strain-level metagenomic profiling. MSYSTEMS. (2017) 2:e00164.16. 10.1128/mSystems.00164-16 28144631 PMC5264247

[22] Mitchell CM Mazzoni C Hogstrom L Bryant A Bergerat A Cher A. Delivery mode affects stability of early infant gut microbiota. Cell Rep Med. (2020) 1:100156. 10.1016/j.xcrm.2020.100156 33377127 PMC7762768

[23] Walker RW Clemente JC Peter I Loos RJF. The prenatal gut microbiome: are we colonized with bacteria in utero? Pediatr Obes. (2017) 12:3–17. 10.1111/ijpo.12217 28447406 PMC5583026

[24] Hyman RW Fukushima M Jiang H Fung E Rand L Johnson B. Diversity of the vaginal microbiome correlates with preterm birth. Reprod Sci. (2014) 21:32–40. 10.1177/1933719113488838 23715799 PMC3857766

[25] Wheeler S Pryor K Antczak B Truong T Murtha A Seed P. The relationship of cervical microbiota diversity with race and disparities in preterm birth. J Neonatal Perinatal Med. (2018) 11:305–10. 10.3233/NPM-17111 30198877

[26] McGregor JA French JI Seo K. Premature rupture of membranes and bacterial vaginosis. Am J Obstet Gynecol. (1993) 169:463–6. 10.1016/0002-9378(93)90342-G 8357046

[27] Witkin SS. The vaginal microbiome, vaginal anti-microbial defence mechanisms and the clinical challenge of reducing infection-related preterm birth. BJOG. (2015) 122:213–8. 10.1111/1471-0528.13115 25316066

[28] Onderdonk AB Delaney ML Fichorova RN. The human microbiome during bacterial vaginosis. Clin Microbiol Rev. (2016) 29:223 LP–38. 10.1128/CMR.00075-15 26864580 PMC4786887

[29] Groer MW Luciano AA Dishaw LJ Ashmeade TL Miller E Gilbert JA. Development of the preterm infant gut microbiome: a research priority. Microbiome. (2014) 2:38. 10.1186/2049-2618-2-38 25332768 PMC4203464

[30] Doyle RM Harris K Kamiza S Harjunmaa U Ashorn U Nkhoma M. Bacterial communities found in placental tissues are associated with severe chorioamnionitis and adverse birth outcomes. PLoS ONE. (2017) 12:e0180167. 10.1371/journal.pone.0180167 28700642 PMC5507499

[31] Prince AL Ma J Kannan PS Alvarez M Gisslen T Harris RA. The placental membrane microbiome is altered among subjects with spontaneous preterm birth with and without chorioamnionitis. Am J Obstet Gynecol. (2016) 214:627.e1–16. 10.1016/j.ajog.2016.01.193 26965447 PMC4909356

[32] Scheiman J Luber JM Chavkin TA MacDonald T Tung A Pham LD. Meta-omics analysis of elite athletes identifies a performance-enhancing microbe that functions via lactate metabolism. Nat Med. (2019) 25:1104–9. 10.1038/s41591-019-0485-4 31235964 PMC7368972

[33] Schönherr-Hellec S Klein GL Delannoy J Ferraris L Rozé JC Butel MJ. Clostridial strain-specific characteristics associated with necrotizing enterocolitis. Appl Environ Microbiol. (2018) 84:e02428–17 10.1128/AEM.02428-17 29352082 PMC5861827

[34] Chernikova DA Koestler DC Hoen AG Housman ML Hibberd PL Moore JH. Fetal exposures and perinatal influences on the stool microbiota of premature infants. J Matern Neonatal Med. (2016) 29:99–105. 10.3109/14767058.2014.987748 25394613 PMC4476945

[35] Sim K Shaw AG Randell P Cox MJ McClure ZE Li MS. Dysbiosis anticipating necrotizing enterocolitis in very premature infants. Clin Infect Dis. (2015) 60:389–97. 10.1093/cid/ciu822 25344536 PMC4415053

[36] Costello EK Carlisle EM Bik EM Morowitz MJ Relman DA. Microbiome assembly across multiple body sites in low-birthweight infants. mBio. (2013) 4:e00782–13 10.1128/mBio.00782-13 24169577 PMC3809564

[37] Reyman M van Houten MA van Baarle D Bosch AATM Man WH Chu MLJN. Impact of delivery mode-associated gut microbiota dynamics on health in the first year of life. Nat Commun. (2019) 10:4997. 10.1038/s41467-019-13373-1 31676793 PMC6825150

[38] Soeorg H Metsvaht T Eelmäe I Merila M Treumuth S Huik K. The role of breast milk in the colonization of neonatal gut and skin with coagulase-negative staphylococci. Pediatr Res. (2017) 82:759–67. 10.1038/pr.2017.150 28665928

[39] Murphy K Curley D O'Callaghan TF O'Shea CA Dempsey EM O'Toole PW. The composition of human milk and infant faecal microbiota over the first three months of life: a pilot study. Sci Rep. (2017) 7:40597. 10.1038/srep40597 28094284 PMC5240090

[40] Forsgren M Isolauri E Salminen S Rautava S. Late preterm birth has direct and indirect effects on infant gut microbiota development during the first six months of life. Acta Paediatr. (2017) 106:1103–9. 10.1111/apa.13837 28316118 PMC5763336

[41] Tauchi H Yahagi K Yamauchi T Hara T Yamaoka R Tsukuda N. Gut microbiota development of preterm infants hospitalised in intensive care units. Benef Microbes. (2019) 10:641–51. 10.3920/BM2019.0003 31179713

[42] Korpela K Blakstad EW Moltu SJ Strømmen K Nakstad B Rønnestad AE. Intestinal microbiota development and gestational age in preterm neonates. Sci Rep. (2018) 8:2453. 10.1038/s41598-018-20827-x 29410448 PMC5802739

[43] Arboleya S Sánchez B Milani C Duranti S Solís G Fernández N. Intestinal microbiota development in preterm neonates and effect of perinatal antibiotics. J Pediatr. (2015) 166:538–44. 10.1016/j.jpeds.2014.09.041 25444008

[44] Biagi E Aceti A Quercia S Beghetti I Rampelli S Turroni S. Microbial community dynamics in mother's milk and Infant's mouth and gut in moderately preterm infants. Front Microbiol. (2018) 9:2512. 10.3389/fmicb.2018.02512 30405571 PMC6204356

[45] Lawson MAE O'neill IJ Kujawska M Gowrinadh Javvadi S Wijeyesekera A Flegg Z. Breast milk-derived human milk oligosaccharides promote Bifidobacterium interactions within a single ecosystem. ISME J. (2020) 14:635–48. 10.1038/s41396-019-0553-2 31740752 PMC6976680

[46] Devika NT Raman K. Deciphering the metabolic capabilities of Bifidobacteria using genome-scale metabolic models. Sci Rep. (2019) 9:18222. 10.1038/s41598-019-54696-9 31796826 PMC6890778

[47] Zhong H Penders J Shi Z Ren H Cai K Fang C. Impact of early events and lifestyle on the gut microbiota and metabolic phenotypes in young school-age children. Microbiome. (2019) 7:2. 10.1186/s40168-018-0608-z 30609941 PMC6320620

[48] Casaburi G Duar RM Brown H Mitchell RD Kazi S Chew S. Metagenomic insights of the infant microbiome community structure and function across multiple sites in the United States. Sci Rep. (2021) 11:1472. 10.1038/s41598-020-80583-9 33479326 PMC7820601

[49] Xiao L Wang J Zheng J Li X Zhao F. Deterministic transition of enterotypes shapes the infant gut microbiome at an early age. Genome Biol. (2021) 22:243. 10.1186/s13059-021-02463-3 34429130 PMC8383385

[50] Zhu D Xiao S Yu J Ai Q He Y Cheng C. Effects of one-week empirical antibiotic therapy on the early development of gut microbiota and metabolites in preterm infants. Sci Rep. (2017) 7:8025. 10.1038/s41598-017-08530-9 28808302 PMC5556106

[51] Zou ZH Liu D Li HD Zhu DP He Y Hou T. Prenatal and postnatal antibiotic exposure influences the gut microbiota of preterm infants in neonatal intensive care units. Ann Clin Microbiol Antimicrob. (2018) 17:9. 10.1186/s12941-018-0264-y 29554907 PMC5858143

[52] Ma J Li Z Zhang W Zhang C Zhang Y Mei H. Comparison of gut microbiota in exclusively breast-fed and formula-fed babies: a study of 91 term infants. Sci Rep. (2020) 10:15792. 10.1038/s41598-020-72635-x 32978424 PMC7519658

[53] Zwittink RD van Zoeren-Grobben D Martin R van Lingen RA Groot Jebbink LJ Boeren S. Metaproteomics reveals functional differences in intestinal microbiota development of preterm infants. Mol Cell Proteomics. (2017) 16:1610–20. 10.1074/mcp.RA117.000102 28684633 PMC5587861

[54] Younge NE Newgard CB Cotten CM Goldberg RN Muehlbauer MJ Bain JR. Disrupted maturation of the microbiota and metabolome among extremely preterm infants with postnatal growth failure. Sci Rep. (2019) 9:8167. 10.1038/s41598-019-44547-y 31160673 PMC6546715

[55] Gómez M Moles L Espinosa-Martos I Bustos G de Vos W Fernández L. Bacteriological and immunological profiling of meconium and fecal samples from preterm infants: a two-year follow-up study. Nutrients. (2017) 9:1293. 10.3390/nu9121293 29186903 PMC5748744

[56] Mshvildadze M Neu J Mai V. Intestinal microbiota development in the premature neonate: establishment of a lasting commensal relationship? Nutr Rev. (2008) 66:658–63. 10.1111/j.1753-4887.2008.00119.x 19019028

[57] Moles L Gómez M Heilig H Bustos G Fuentes S de Vos W. Bacterial diversity in meconium of preterm neonates and evolution of their fecal microbiota during the first month of life. PLoS ONE. (2013) 8:e66986. 10.1371/journal.pone.0066986 23840569 PMC3695978

[58] Gregory KE Samuel BS Houghteling P Shan G Ausubel FM Sadreyev RI. Influence of maternal breast milk ingestion on acquisition of the intestinal microbiome in preterm infants. Microbiome. (2016) 4:68. 10.1186/s40168-016-0214-x 28034306 PMC5200970

[59] Parra-Llorca A Gormaz M Alcántara C Cernada M Nuñez-Ramiro A Vento M. Preterm gut microbiome depending on feeding type: significance of donor human milk. Front Microbiol. (2018) 9:1376. 10.3389/fmicb.2018.01376 29997594 PMC6030370

[60] Chernikova DA Madan JC Housman ML Zain-ul-abideen M Lundgren SN Morrison HG. The premature infant gut microbiome during the first 6 weeks of life differs based on gestational maturity at birth. Pediatr Res. (2018) 84:71–9. 10.1038/s41390-018-0022-z 29795209 PMC6082716

[61] Walter J. Ecological role of lactobacilli in the gastrointestinal tract: implications for fundamental and biomedical research. Appl Environ Microbiol. (2008) 74:4985–96. 10.1128/AEM.00753-08 18539818 PMC2519286

[62] Pärtty A Luoto R Kalliomäki M Salminen S Isolauri E. Effects of early prebiotic and probiotic supplementation on development of gut microbiota and fussing and crying in preterm infants: a randomized, double-blind, placebo-controlled trial. J Pediatr. (2013) 163:1272–7.e2. 10.1016/j.jpeds.2013.05.035 23915796

[63] Alcon-Giner C Dalby MJ Caim S Ketskemety J Shaw A Sim K. Microbiota supplementation with bifidobacterium and lactobacillus modifies the preterm infant gut microbiota and metabolome: an observational study. Cell Rep Med. (2020) 1:100077. 10.1016/j.xcrm.2020.100077 32904427 PMC7453906

[64] Davis EC Dinsmoor AM Wang M Donovan SM. Microbiome composition in pediatric populations from birth to adolescence: impact of diet and prebiotic and probiotic interventions. Dig Dis Sci. (2020) 65:706–22. 10.1007/s10620-020-06092-x 32002758 PMC7046124

[65] Radmacher PG Adamkin DH. Fortification of human milk for preterm infants. Semin Fetal Neonatal Med. (2017) 22:30–5. 10.1016/j.siny.2016.08.004 27593561

[66] Zhang L van Dijk ADJ Hettinga K. An interactomics overview of the human and bovine milk proteome over lactation. Proteome Sci. (2016) 15:1. 10.1186/s12953-016-0110-0 28149201 PMC5267443

[67] Anil KC Basel PL Singh S. Low birth weight and its associated risk factors: Health facility-based case-control study. PLoS ONE. (2020) 15:e0234907. 10.1371/journal.pone.0234907 32569281 PMC7307746

[68] Karlberg J Albertsson-Wikland K. Growth in full-term small-for-gestational-age infants: from birth to final height. Pediatr Res. (1995) 38:733–9. 10.1203/00006450-199511000-00017 8552442

[69] Martin A Connelly A Bland RM Reilly JJ. Health impact of catch-up growth in low-birth weight infants: systematic review, evidence appraisal, and meta-analysis. Matern Child Nutr. (2017) 13. 10.1111/mcn.12297 27002681 PMC7158701

[70] Roswall J Olsson LM Kovatcheva-Datchary P Nilsson S Tremaroli V Simon MC. Developmental trajectory of the healthy human gut microbiota during the first 5 years of life. Cell Host Microbe. (2021) 29:765–6.e3. 10.1016/j.chom.2021.02.021 33794185

