# Supplementary material for: Human Milk-Based or Bovine Milk-Based Fortifiers Differentially Impact the Development of the Gut Microbiota of Preterm Infants
Source: Front Pediatr. 2021 Nov 30;9:719096. doi: 10.3389/fped.2021.719096 (PMC8669825; doi:10.3389/fped.2021.719096)
Supplement: Supplementary file 1 [file Data_Sheet_1.docx]

Supplementary Material

# Supplementary Tables

**Supplementary Table 1.** Milk Consumption* over hospitalization period

| **Corrected GA** | **MOM** | **DHM** | **HM** | **Formula** |
| --- | --- | --- | --- | --- |
| **25 wks** | 100 | - | 100 | - |
| **26 wks** | 86.0 ± 12.4 | 14.0 ± 12.4 | 100 | - |
| **27 wks** | 81.0 ± 37.8 | 19.0 ± 37.8 | 100 | - |
| **28 wks** | 81.4 ± 34.4 | 18.6 ± 34.4 | 100 | - |
| **29 wks** | 81.3 ± 34.3 | 18.7 ± 34.3 | 100 | - |
| **30 wks** | 60.1 ± 41.1 | 39.9 ± 41.1 | 100 | - |
| **31 wks** | 63.9 ± 39.9 | 35.1 ± 38.3 | 99.1 ± 5.88 | 0.9 ± 5.88 |
| **32 wks** | 63.4 ± 40.5 | 35.6 ± 39.5 | 99.0 ± 6.73 | 1.0 ± 6.73 |
| **33 wks** | 70.4 ± 38.1 | 27.2 ± 36.2 | 97.6 ± 10.6 | 2.4 ± 10.6 |
| **34 wks** | 68.5 ± 42.9 | 20.7 ± 32.1 | 89.2 ± 18.7 | 10.8 ± 18.7 |
| **35 wks** | 72.3 ± 41.3 | 4.8 ± 15.4 | 77.1 ± 38.6 | 22.9 ± 38.6 |
| **36 wks** | 72.2 ± 41.4 | - | 72.2 ± 41.4 | 27.8 ± 41.4 |
| **37 wks** | 64.8 ± 46.2 | - | 64.8 ± 46.2 | 35.2 ± 46.2 |
| **38 wks** | 73.0 ± 43.5 | - | 73.0 ± 43.5 | 27.0 ± 43.5 |
| **39 wks** | 98.5 ± 3.39 | - | 98.5 ± 3.39 | 1.5 ± 3.39 |
| **40 wks** | 46.4 ± 53.9 | - | 46.4 ± 53.9 | 53.6 ± 53.9 |
| *Percentage of total feeding volume  Data presented as mean ± SD.  Abbreviations: GA: gestational age; MOM: mother’s own milk; DHM: donor human milk; HM: human milk (MOM+DHM) | | | | |

**Supplementary Table 2**. Alpha diversity indices and perinatal factors

| **Characteristic** | **Observed OTUs** | | **Shannon Index** | |
| --- | --- | --- | --- | --- |
|  | mean ± SD | p* | mean ± SD | p* |
| **Maternal Race** |  |  |  |  |
| Black | 23.3 ± 10.3 | 0.0026 | 2.14 ± 1.10 | 0.55 |
| White | 17.5 ± 11 |  | 2.04 ± 0.91 |  |
| **Chorioamnionitis** |  |  |  |  |
| Positive | 18.6 ± 8.90 | 0.46 | 2.34 ± 0.79 | 0.13 |
| Negative | 19.1 ± 11.6 |  | 2.01 ± 0.98 |  |
| **PROM** |  |  |  |  |
| Positive | 18.0 ± 9.16 | 0.57 | 2.06 ± 1.02 | 0.93 |
| Negative | 19.4 ± 11.8 |  | 2.09 ± 0.79 |  |
| **Infant Sex** |  |  |  |  |
| Female | 18.4 ± 8.92 | 0.86 | 1.98 ± 0.88 | 0.29 |
| Male | 19.9 ± 14.0 |  | 2.22 ± 1.08 |  |
| **Mode of delivery** |  |  |  |  |
| Vaginal | 18.2 ± 8.51 | 0.48 | 2.18 ± 0.94 | 0.36 |
| C-Section | 19.2 ± 11.7 |  | 2.04 ± 0.97 |  |
| **Mechanical Ventilation** |  |  |  |  |
| Positive | 19.6 ± 18.8 | 0.17 | 2.18 ± 1.15 | 0.19 |
| Negative | 19.6 ± 10.5 |  | 2.03 ± 0.89 |  |
| **Use of Antibiotics** |  |  |  |  |
| Positive | 15.8 ± 9.82 | 0.32 | 1.91 ± 1.2 | 0.53 |
| Negative | 19.9 ± 11.3 |  | 2.11 ± 0.88 |  |
| Data presented as mean ± SD.  *Differences based on GLM analysis, adjusting for corrected GA and antibiotic use.  Abbreviations: PROM: premature rupture of membranes. | | | | |

**Supplementary Table 3.** Differences in Unweighted and Weighted UniFrac Distances in the early-life microbiota of PT infants.

| **Characteristic** | **Unweighted Unifrac Distances** | | **Weighted UniFrac Distances** | |
| --- | --- | --- | --- | --- |
|  | R^2^ | p* | R^2^ | p* |
| **Maternal Race** | 0.095 | 0.012 | 0.045 | 0.021 |
| **Chorioamnionitis** | 0.13 | 0.011 | 0.13 | 0.01 |
| **PROM** | 0.004 | 0.023 | 0.001 | 0.023 |
| **Mode of Delivery** | 0.31 | 0.008 | 0.31 | 0.008 |
| **Mechanical Ventilation** | 0.10 | 0.013 | 0.096 | 0.013 |
| **Infant Sex** | 0.006 | 0.023 | 0.004 | 0.023 |
| **Corrected GA** | 0.26 | 0.009 | 0.28 | 0.009 |
| **Use of Antibiotics** | 0.57 | 0.013 | 0.56 | 0.013 |
| *Differences based on Adonis test with 999 permutations.  Abbreviations: PROM: premature rupture of membranes; GA: gestational age. Corrected GA: GA at birth + postnatal age. | | | | |

**Supplementary Table 4.** Differences in Unweighted and Weighted UniFrac Distances over the course of hospitalization in the NICU

| **Characteristic** | **Unweighted Unifrac Distances** | | **Weighted UniFrac Distances** | |
| --- | --- | --- | --- | --- |
|  | R^2^ | p* | R^2^ | p* |
| **Infant** | 0.47 | 0.001 | 0.55 | 0.001 |
| **Maternal Race** | 0.009 | 0.001 | 0.009 | 0.019 |
| **Chorioamnionitis** | 0.007 | 0.001 | 0.013 | 0.007 |
| **PROM** | 0.004 | 0.025 | 0.0006 | 0.69 |
| **Mode of Delivery** | 0.005 | 0.005 | 0.012 | 0.004 |
| **Mechanical Ventilation** | 0.005 | 0.009 | 0.003 | 0.18 |
| **Infant Sex** | 0.004 | 0.03 | 0.0021 | 0.31 |
| **Postnatal age** | 0.044 | 0.001 | 0.10 | 0.003 |
| **Corrected GA** | 0.006 | 0.002 | 0.012 | 0.008 |
| **Use of Antibiotics** | 0.003 | 0.046 | 0.002 | 0.31 |
| *Differences based on Adonis test with 999 permutations.  Abbreviations: PROM: premature rupture of membranes; GA: gestational age. Corrected GA: GA at birth + postnatal age. | | | | |

**Supplementary Table 4**. Age, growth, use of antibiotic use and dietary distribution of samples before and after enterotype change.

| **Characteristic** | **Before**  **n=113** | **After**  **n=143** | **p*** |
| --- | --- | --- | --- |
| **Corrected GA, wks** | 31.7 ± 2.34 | 34.7±2.3 | <0.001 |
| **Weight, g** | 1578 ± 590 | 2091±599 | <0.001 |
| **Antibiotic use, n-%** | 11 - 9.73 | 4-2.79 | 0.037 |
| **Type of Diet, n-%** |  |  |  |
| HM | 3 - 2.65 | 3 - 2.10 | <0.001 |
| HM + HMF | 24 - 21.2 | 6 - 4.19 |  |
| HM + BMF | 82 - 72.6 | 102 - 71.3 |  |
| HM + Both | 4 - 3.53 | 5 - 3.49 |  |
| Formula + BMF | 0 | 4 - 2.79 |  |
| Formula | 0 | 23 - 16.1 |  |
| Data presented as n - %, or mean ± sd.  Abbreviations: GA: gestational age; HM: human milk; HMF: human milk-based fortifier, BMF: bovine milk-based fortifier  *Differences based on chi-square test for categorical variables and Kruskal-Wallis for numerical variables | | | |

# Supplementary Figures


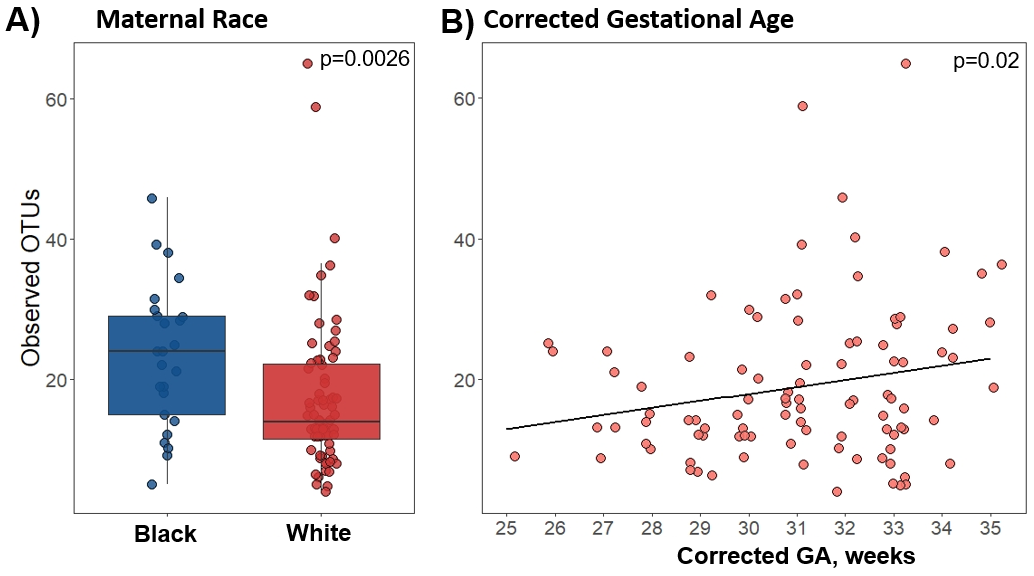


**Supplementary Figure 1.** Factors associated with alpha diversity after birth. Maternal race was significantly associated with the number of OTUs. Infants from non-white mothers had higher number of OTUs compared to infants of white mothers (A). There was a positive associated between corrected gestational age and number of observed OTUs (B).


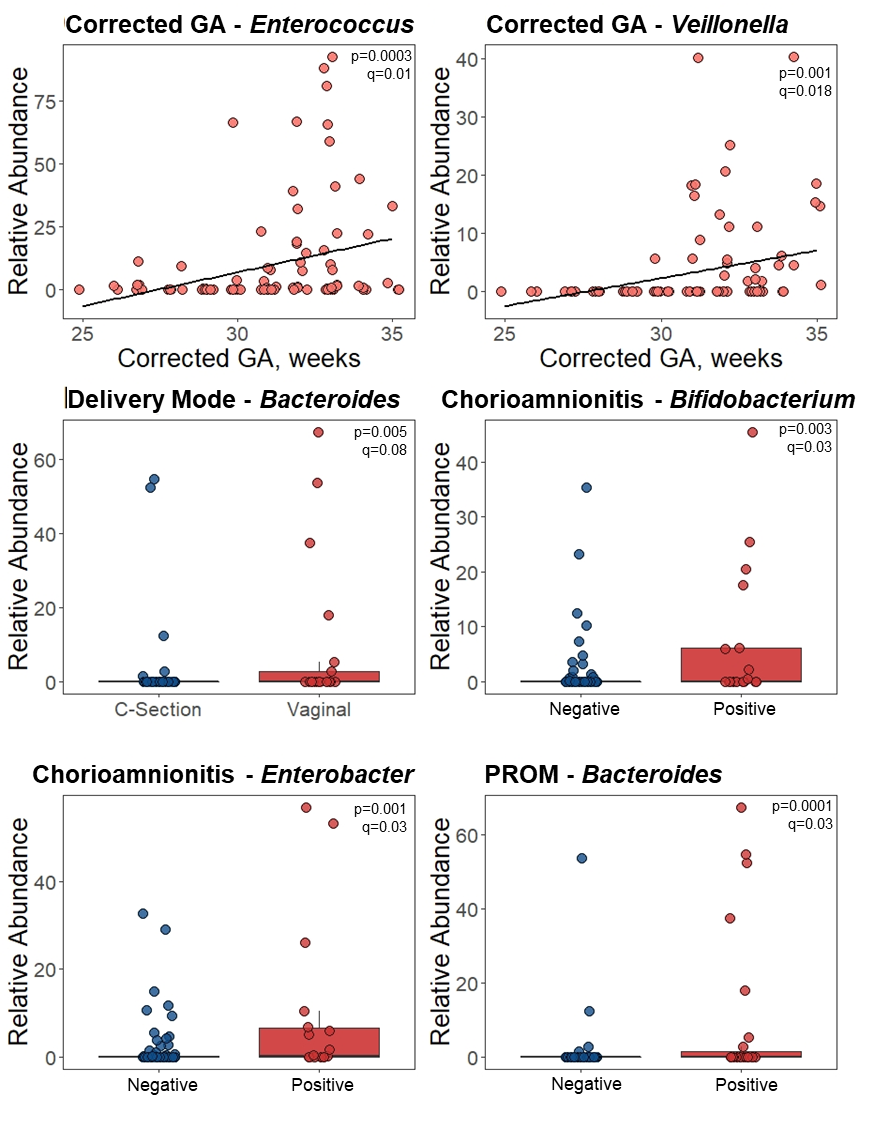


**Supplementary Figure 2.** Differences in genera after birth based on perinatal factors.

After birth (~2.16 weeks postnatal age, ~31.1 weeks corrected GA), there was a positive association between the abundance of *Enterococcus* and *Veillonella* with corrected GA (q=0.01 and q=0.018, respectively). Infants exposed to Chorioamnionitis had greater abundances of *Bifidobacterium* and *Enterobacter* (q=0.03 both). Premature rupture of membranes was associated with increase abundance of *Bacteroides* (q=0.03). Infants vaginally delivered tended to have (q<0.1) higher abundance of *Bacteroides* compared to those born via C-Section.


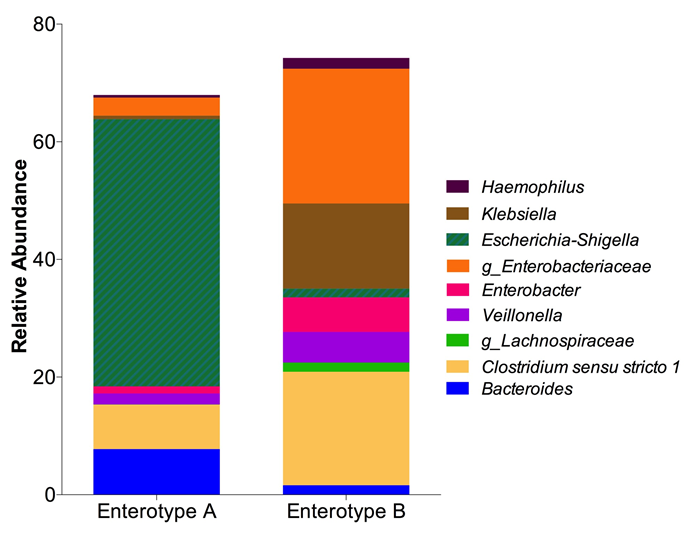


**Supplementary Figure 3.** Enterotypes taxonomic composition. Relative abundance of bacteria were significantly different between the enterotypes.


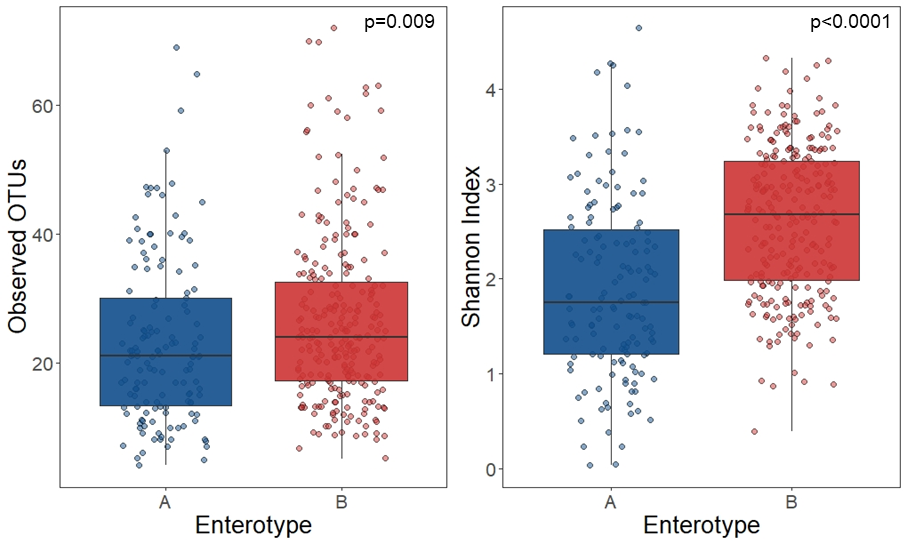


**Supplementary Figure 4.** Alpha diversity differences between enterotypes. Boxplots representing alpha diversity indices (Observed OTUs and Shannon Index). Enterotype B had significantly higher alpha diversity indices compared to Enterotype A.
